# Modeling Water Interactions with Graphene and Graphite via Force Fields Consistent with Experimental Contact Angles

**DOI:** 10.1021/acs.jpclett.4c01143

**Published:** 2024-06-10

**Authors:** Shane R. Carlson, Otto Schullian, Maximilian R. Becker, Roland R. Netz

**Affiliations:** † Fachbereich Physik, Freie Universität Berlin, Arnimallee 14, D-14195 Berlin, Germany; ‡ Department of Biomaterials, Max Planck Institute of Colloids and Interfaces, D-14424 Potsdam, Germany

## Abstract

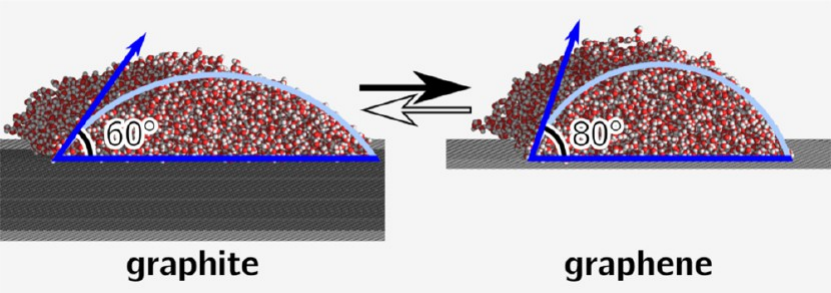

Accurate simulation models for water interactions with graphene and graphite are important for nanofluidic applications, but existing force fields produce widely varying contact angles. Our extensive review of the experimental literature reveals extreme variation among reported values of graphene–water contact angles and a clustering of graphite–water contact angles into groups of freshly exfoliated (60° ± 13°) and not-freshly exfoliated graphite surfaces. The carbon–oxygen dispersion energy for a classical force field is optimized with respect to this 60° graphite–water contact angle in the infinite-force-cutoff limit, which in turn yields a contact angle for unsupported graphene of 80°, in agreement with the mean of the experimental results. Interaction force fields for finite cutoffs are also derived. A method for calculating contact angles from pressure tensors of planar equilibrium simulations that is ideally suited to graphite and graphene surfaces is introduced. Our methodology is widely applicable to any liquid-surface combination.

Graphene has been the subject of intense interest and research for decades now due to its remarkable combination of characteristics, including optical transparency, electrical conductivity, and mechanical strength.^1−5^ It shows immense promise for various nanoscience applications, including DNA sequencing,^6^ energy storage,^7^ and filtration.^8^ Its exceptional mechanical, thermal, and chemical stability, and impermeability to gases make it promising for advanced coating applications, which is of particular relevance here.^9−12^ In order to fully harness the potential of graphene, a deep understanding of its interactions with liquids, in particular water, is imperative. Wetting is a fundamental characteristic that describes the interaction between a liquid and a surface. Its importance extends across various scientific, technological, and industrial fields, particularly in areas like nano- and microfluidics.^13−15^ For graphene in particular, the strength of its interaction with water is of key importance for numerous applications as it directly influences properties such as charge doping,^16^ carrier mobility,^17^ and adhesion.^18^ It can influence the energy storage capacity of graphene supercapacitors^19^ and the heat exchange between graphene-coated copper substrates and water vapor.^20^ It is therefore of great practical value to gain a comprehensive understanding and develop the tools necessary to model these effects correctly.

Some works suggest that graphene should exhibit complete transparency to electrostatic and dispersive interactions between adsorbed water above and the underlying substrate below,^20,21^ except when it is contaminated or corrugated,^21^ or when the adsorption occurs through short-range polar bonds.^20^ Contrasting evidence, however, suggests the absence of any interaction transparency in graphene,^22^ while yet other studies propose that it exhibits only partial transparency.^23−25^ The resolution of this issue remains experimentally elusive, partly due to the difficulty of measuring contact angles on clean and pure graphene. The effects of both airborne^25,26^ and solvent-induced^27,28^ contamination can be significant, and have proven to be difficult to eliminate, an effect also seen in other two-dimensional materials.^29^ Another complication is the thinness of graphene, which necessitates support from a substrate. Although inventive methods have been employed recently, such as floating graphene on water and trapping air bubbles underneath,^30^ utilizing hydrogel as a floating substrate,^21^ or partially suspending graphene on nanotextured substrates,^31^ experimental difficulties persist. This is especially relevant as graphene has kindled interest in a profusion of other two-dimensional materials and material combinations where the same issues will arise.^29^

Our objective is to accurately model water interactions with graphene and graphite by developing force fields for molecular dynamics simulation that are optimized with respect to the best experimental data available. Our methods are general and can be applied to any liquid and substrate material. Because interactions between carbon and water oxygen in the infinite-force-cutoff limit are pairwise and additive, we propose that a single nonpolarizable force field that accurately reproduces the experimental wetting behavior of both graphene and graphite simultaneously is feasible, under the assumption of the interaction transparency of graphene sheets.

Contact angles can be measured directly from simulations of liquid droplets on surfaces, where, to eliminate finite-size effects by extrapolation, droplet size is varied.^32−34^ Alternatively, the work of adhesion can be obtained by thermodynamic integration^35^ or phantom wall methods.^36,37^ A recent approach consists of indirect umbrella sampling in capillary channels with applied biasing potentials.^38^ One key commonality among these methods is that they require many specialized simulations. We propose an alternative approach, ideally suited for use on graphenic systems, based on work by Sedlmeier et al.^39^ and Sendner et al.^40^ where interfacial tensions are calculated using just the pressure tensors of planar simulations. This method has the advantage of requiring at most three simple planar equilibrium simulations, making simulation setup facile.

We begin by conducting an extensive literature review to compile experimentally measured contact angles of graphene (see Supporting Information Section S1). Figure 1 (a) shows the distribution of these contact angle results. Note that the measurements conducted by Prydatko et al.^30^ (red) stand out from the rest. They employ an “inverted” system where graphene is floated on an air–water interface, and measure the contact angle of an air bubble trapped underneath. All other experiments use sessile water droplets.^10,20−23,25,30,31,41−46^ One notable measurement by Ondarcuhu et al.^31^ attempts to minimize the substrate influence by partially suspending the graphene sheet, resulting in a contact angle of 85° ± 5°. The contact angle values of sessile droplets as a whole span a wide range, from 10° to 140° with an average of 74° ± 35°, which might arise from, among other things, contamination, the use of different substrates in the measurements and/or wetting transparency. This wide variation is, in any case, crucial information, as it makes clear that optimizing a force field with respect to any particular experimental graphene contact angle is inadvisible, absent further information.

**Figure 1:**
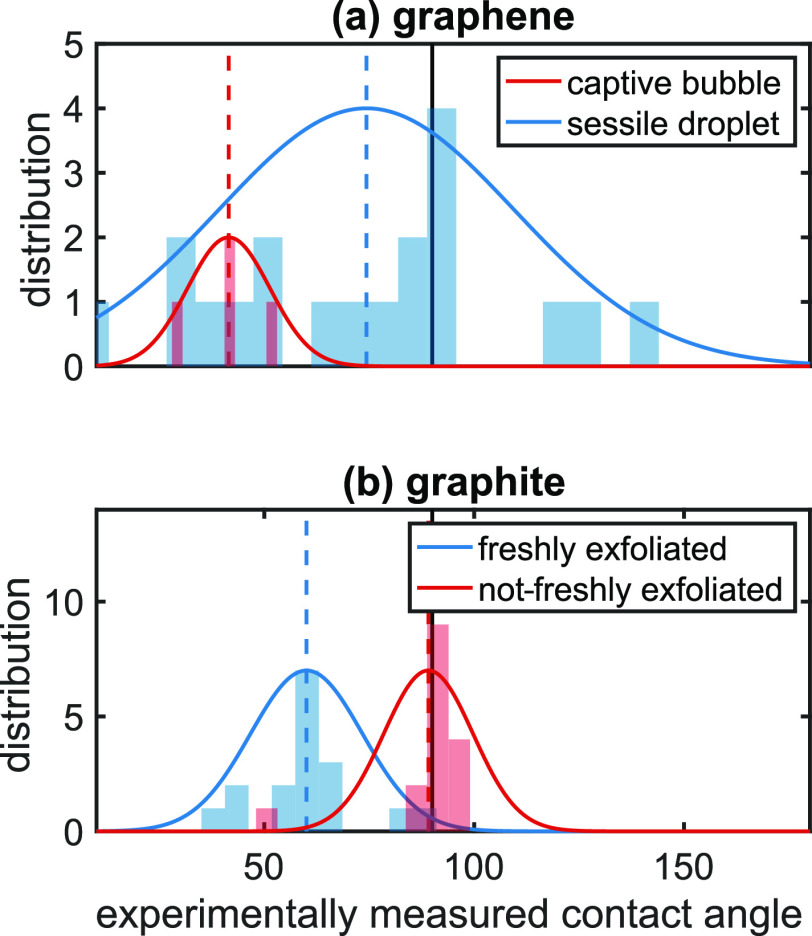
Histograms of experimentally measured contact angles for water on graphene and graphite. Also plotted are Gaussian functions with the mean and standard deviation of the sample distributions. (a) Graphene: the average value for captive bubbles is 41.5° ± 9.8° and for sessile droplets 74° ± 35°. (b) Graphite: the average value for freshly exfoliated graphite is 60° ± 13°, and for not-freshly exfoliated graphite, 89° ± 10°. The black vertical line at 90° separates the hydrophobic and hydrophilic regimes.

The unreliability of experimental graphene–water contact angles leads us to instead optimize carbon–water interaction potentials with respect to experimental graphite–water contact angles, so we also conduct a literature review of these (see Supporting Information Section S1). We categorize the graphite–water experimental results into two groups: measurements on freshly exfoliated^21,25,26,30,31,43,47−52^ and not-freshly exfoliated surfaces.^10,20,22,25,26,31,42,45,50−54^ Here, not-freshly exfoliated graphite includes all experimental values where the surface preparation is not specified, as well as exfoliated surfaces that are left to sit for an allotted period before measurement. Figure 1 (b) shows the distributions of the measured contact angles. The two distributions, for freshly exfoliated and not-freshly exfoliated surfaces, cluster visibly around two distinct mean values. The not-freshly exfoliated surfaces exhibit contact angles near the hydrophobic/hydrophilic threshold, with an average contact angle of 89° ± 10°, while the freshly exfoliated graphite surfaces are significantly more hydrophilic, with an average contact angle of 60° ± 13°. It is worth noting that contact angles significantly below 90° were observed for graphite as early as 1975,^48^ and specific force fields were developed for such systems, but these have not been extensively utilized. There is clear evidence that the increase in contact angle over time for exfoliated graphite is due to surface contamination by airborne hydrocarbons, both from recent experimental^25^ and simulation^26^ studies. In this light, we adopt the contact angle of freshly exfoliated graphite as the target value for our graphene-/graphite–water force field.

We have compiled a comprehensive list of currently used force fields that model the interaction between graphene or graphite and water solely through a Lennard-Jones (LJ) potential between carbon and oxygen atoms. The force fields, organized by their hydrophobicity, are presented in Table 1, which gives the contact angle values they were developed to target, where applicable. Importantly, except for the force field developed by Werder et al.,^55^ all force fields targeting specific contact angles, target ones significantly greater than the experimental average of 60° ± 13° we find for freshly exfoliated graphite.

**Table 1:** Force Fields from Literature Sorted by Increasing Hydrophobicity

Ref	ϵ_CO_ [kJ/mol]	σ_CO_ [nm]	Contact angle
Werder et al.^55^	0.5643	0.319	target (graphite): 42°
Hummer et al.^56^	0.477	0.328	graphene: 57° (TIP3P^57^)^58^
this work	0.3807	0.3367	target (graphite): 60°
			graphene: 80° (SPC/E, ∞ nm)
			graphite: 60° (SPC/E, ∞ nm)
this work	0.4391	0.3367	target (graphene): 80° (SPC/E, 0.9 nm)
this work	0.5164	0.3367	target (graphite): 60° (SPC/E, 0.9 nm)
Won et al.^59^^a^	0.4339	0.3278	graphite: 69.1°^60^
GROMOS53a6^61^	0.4247	0.3367	graphene: 82.74° (SPC/E,^62^ 0.9 nm)^63^
			graphite: 78.42° (SPC/E, 0.9 nm)^63^
Werder et al.^55^	0.392	0.319	target (graphite): 86°
			graphene: 95° (SPC/E)^58^
			graphite: ∼90° (SPC/E, 2 nm)^22^
Liao et al.^64^	0.3998	0.319	target (graphene): 92.5°
			graphene: 91.83° (SPC/E)^64^
Jaffe et al.^65^	0.357	0.319	target (graphite): 95°
			graphene: 100.7° (SPC/E, 2 nm)^66^
			graphite: 90.2° (SPC/E, 2 nm)^66^
Bejagam et al.^67^	0.32505	0.3282746	target (graphene): 97.5°
Tummala et al.^68^	0.3892	0.3283	graphene: 104°^23^
Scocchi et al.^69^	0.200	0.319	target (graphene): 127°
			graphite: 129.9° ^60^
Taherian et al.^66^	0.205	0.319	target (graphene): 127°
			graphite: 127.0° (SPC/E, 2 nm)^66^

a Taken from ref (60).

Among the force fields that were not developed to reproduce a specific contact angle, the one utilized by Hummer et al.^56^ is closest to reproducing the 60° graphite contact angle. However, this force field is excessively hydrophilic, giving a graphene–water contact angle of 57° when used with a LJ cutoff of 1 nm (the graphite–water contact angle will be lower due to the additional sheets of graphene that interact with the water). There are also force fields in use where no specific contact angle data has been reported, such as the AMBER96 force field^70^ employed in a study by Pascal et al.^71^

Several force fields also incorporate additional interaction potentials between water hydrogen and carbon atoms.^60,71^ However, our goal is to develop the simplest force field that can accurately reproduce the experimental wetting behavior of both graphene and graphite without significantly increasing computational cost, so we follow the convention of setting water−hydrogen LJ parameters to zero. In the same vein, we employ fixed-charge models, as the addition of polarizability in graphene−water models has been found not to significantly influence wetting behavior when no electric field is applied.^72^ This finding is supported by the results of Loche et al.,^63^ who observe that the metallic properties of a single graphene sheet have minimal influence on the water density profile and orientation at the surface.

In this work, classical molecular dynamics (MD) simulations are performed using GROMACS 2022^73,74^ to investigate the contact angle of SPC/E water on spatially frozen, nonpolarizable graphene and graphite. Previous studies have shown agreement between contact angles on flexible and spatially frozen graphene.^55,64^ Here, we focus on the influence of the LJ cutoff, interaction strength ϵ_CO_, and number of graphene sheets. The simulations are of two types: planar systems and droplet simulations. Planar systems are translationally invariant in the *xy*-plane and consist of either a pure water film in vacuum (for determining water surface tension), which forms a water vapor phase, or graphene sheets uniformly covered by a water film under a vacuum/water vapor phase. Droplet simulations consist of graphene sheets and water in the form of a cylindrical droplet continuous over the periodic boundary in one direction, also under a vacuum/water vapor phase. Figure 2 (a) and (b) show simulation snapshots of water droplets on a single graphene sheet and on graphite, respectively. Although the macroscopic contact angles of cylindrical and spherical droplets are identical, cylindrical droplets are less sensitive to finite-size effects.^33,34^ Detailed simulation parameters are provided in the Supporting Information Section S2. Traditionally, the determination of contact angles from cylindrical droplets has relied on two-dimensional density distributions.^32−34,55,75−77^ For this work, a faster method for contact angle extraction has been developed: the one-dimensional liquid mass distribution along the surface normal is extracted and fitted with a function based on an integrated radial sigmoid function and the density profile from a planar liquid–solid simulation, which vastly reduces computational complexity and reduces postprocessing time by orders of magnitude (see Supporting Information Section S3).

**Figure 2:**
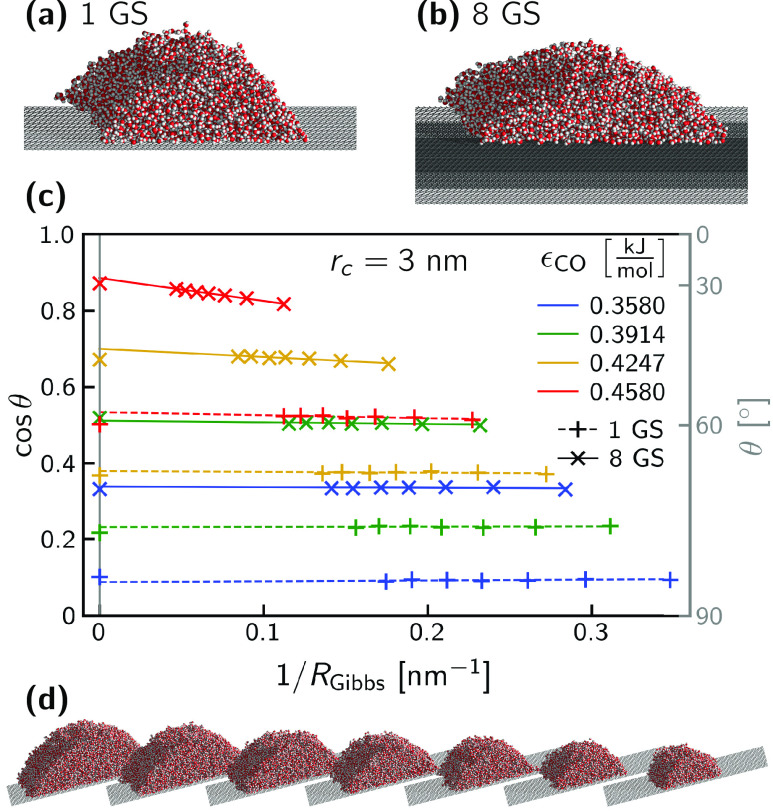
(a, b) Simulation snapshots of cylindrical water droplets on graphene and graphite with ϵ_CO_ = 0.4247 kJ/mol and *r*_*c*_ = 3 nm. (c) Cosines of contact angles θ for finite droplet radii *R*_Gibbs_ extracted from droplet simulations with *r*_*c*_ = 3 nm (data points). Linear fits of cos θ over 1/*R*_Gibbs_ (lines) are extrapolated to 1/*R*_Gibbs_ = 0, giving θ_∞_. Also plotted at 1/*R*_Gibbs_ = 0, for comparison to the linear extrapolations, are cos θ_∞_ values determined from the pressure tensors of planar simulations. Estimated errors are smaller than the data points and are not shown. (d) Snapshots of the differently sized droplets for the 1-graphene-sheet (1 GS) system with ϵ_CO_ = 0.4247 kJ/mol and *r*_*c*_ = 3 nm (gold +’s in (c)).

By determining the microscopic contact angle θ for droplets of different sizes (in our case, parametrized by the radius of the curved liquid–vapor interface of the cylindrical droplet *R*_Gibbs_), as shown in Figure 2 (d), the macroscopic contact angle θ_∞_ can be found. This is necessary because of the significant influence of Tolman corrections to the liquid surface tension, particularly in small systems. The deviations can be well described by a linear dependence of cos θ on 1/*R*_Gibbs_, which enables a linear extrapolation to the macroscopic contact angle.^33,34,55^Figure 2 (c) shows extrapolations for a cutoff of *r*_*c*_ = 3 nm, a range of ϵ_CO_, and either one or eight graphene sheets. The size-dependence due to the Tolman correction can be seen most clearly in the negative slope of cos θ versus 1/*R*_Gibbs_ for more hydrophilic systems (e.g., ϵ_CO_ = 0.4580 kJ/mol, 8 GS), in agreement with previous observations.^32,33^ Linear fits of cos θ over 1/*R*_Gibbs_ are extrapolated to 1/*R*_Gibbs_ = 0, giving the macroscopic contact angle θ_∞_. In addition, macroscopic contact angles determined independently from the pressure tensors of planar simulations are plotted for comparison at 1/*R*_Gibbs_ = 0 and agree well with the extrapolated droplet simulation results.

Figure 3 (a–d) plots macroscopic contact angles θ_∞_ over interaction strength ϵ_CO_, for different cutoffs. For the results in Figure 3 (a), the potential defined in the SPC/E water model was used, which features a LJ cutoff of 0.9 nm with a potential shift. For the results shown in Figure 3 (b–d) meanwhile, a force-switching scheme between *r*_*c*_ – 0.1 nm and *r*_*c*_ was used (see Supporting Information Section S4). For each cutoff, only the nearest ⌊*r*_*c*_/(0.34 nm)⌋ sheets of graphene are simulated, since only they are close enough to interact with the water. Contact angles decrease monotonically with increasing values of ϵ_CO_, cutoff length *r*_*c*_, and number of graphene sheets. This behavior is expected as these parameters enhance attractive interactions between water oxygen and carbon atoms, increasing hydrophilicity. In the Supporting Information Section S5, we provide the parameters for fits of the form
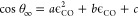
1to describe the curves in Figure 3 (a–d), which can be used to obtain contact angles over the entire investigated range of ϵ_CO_.

**Figure 3:**
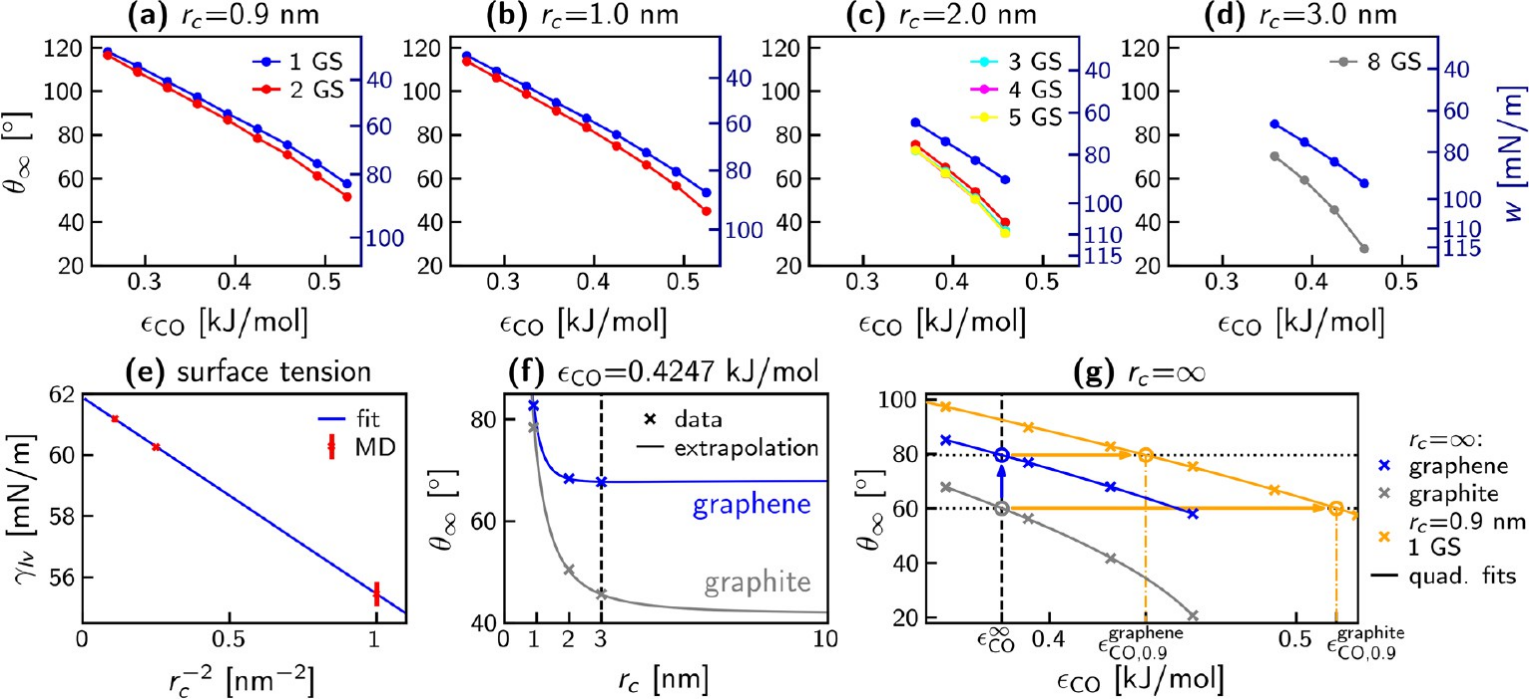
(a–d) Macroscopic contact angles θ_∞_ plotted over ϵ_CO_ (σ_CO_ = 0.3367 nm) for different LJ cutoff lengths *r*_*c*_ and number of graphene sheets (GS). The right-hand *y*-axis on each panel shows the corresponding areal work of adhesion *w* (see eq 3). In (a) a potential-shift at the cutoff is used, while in (b–d), a force-switching scheme between *r*_*c*_ – 0.1 nm and *r*_*c*_ is used. Errors are all smaller than the size of the plotted data points. (e) Surface tension of the water liquid–vapor interface as a function of the inverse square of the cutoff length, for the systems using the force-switching scheme, alongside a linear fit. (f) Analytical extrapolation (eq 4, solid lines) of θ_∞_(*r*_*c*_), fitted to the values from (d) (*r*_*c*_ = 3 nm, indicated by the dashed line) and compared to values from (b) and (c), for ϵ_CO_ = 0.4247 kJ/mol. The asymptotic limits of these fits toward the right give the *r*_*c*_ = ∞ values of θ_∞_. (g) Contact angle as a function of ϵ_CO_ for infinite cutoff for graphene and graphite (blue and gray × ’s), based on data from (d), extrapolated using the method shown in (f), along with fits of eq 1 (solid lines). The vertical dashed line at ϵ_CO_ = 0.3807 kJ/mol (denoted  shows where the infinite-cutoff graphite fit matches the targeted experimental value of 60° (lower dotted black line), which in turn gives a contact angle of 80° for graphene (upper dotted black line). In addition, θ_∞_ for the *r*_*c*_ = 0.9 nm cutoff for a single sheet of graphene is shown (orange, same as blue data in (a)), with a fit of eq 1. The intersections with the black dotted lines give the values  = 0.5164 kJ/mol for graphite and  = 0.4391 kJ/mol for graphene, when *r*_*c*_ = 0.9 nm is used.

To determine the contact angle for free-standing graphene, it is necessary to obtain the contact angle for graphite in the infinite cutoff limit. This is because, in contrast to bulk properties, the interfacial properties of a liquid depend strongly on the cutoff length.^34^ Indeed, longer cutoffs provide better agreement with experimental results for interfacial properties.^34^ This suggests the use of Lennard-Jones Particle Mesh Ewald (LJPME) for long-range LJ forces. However, we found that for LJPME to be accurate, extreme computational expense is needed (see Supporting Information Section S6), and chose instead to extrapolate the finite-cutoff data to the infinite-cutoff limit.

As a preliminary step, we fit the interfacial tension of the water liquid–vapor interface γ_*lv*_ (see the Supporting Information Section S7) as a function of the cutoff length, which is shown in Figure 3 (e), and gives
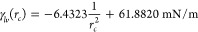
2The  dependence here is based on a previous derivation that shows that the interaction energy of a surface governed by LJ interactions scales with  to leading order.^34^

To extrapolate the contact angle from a finite cutoff to an infinite cutoff, we employ the Young-Dupré equation,^54^ which relates the areal work of adhesion *w* of a liquid phase adsorbed on a surface to its contact angle,
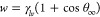
3Formally, changing the cutoff length from *r*_*c*_ to a different  represents a change of the potential, and the related change in free energy can be determined via thermodynamic integration, which is shown in the Supporting Information Section S8 to also work remarkably well for other changes to the free energy, i.e., via ϵ_CO_ and number of graphene sheets. This results in a convenient formula for extrapolating between contact angles for different values of *r*_*c*_ ,
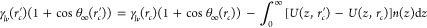
4where *n*(*z*) denotes the number density of liquid molecules and *U*(*z*, *r*_*c*_) the per-molecule interaction potential between the liquid and solid, which is a function of the height *z* from the solid surface and the cutoff *r*_*c*_ (see Supporting Information Section S8). In the case of graphene, *U*(*z*, *r*_*c*_) takes the form , which is the interaction energy of a single water molecule with graphene treated as a continuous sheet with uniform density (see the Supporting Information Section S4). In the case of graphite, *U*(*z*, *r*_*c*_) takes the form  where *z*_*l*_ is the position of the graphene sheet indexed by *l*.

Starting from *r*_*c*_ = 3 nm (a single data point in Figure 3 (d)), we use eq 4 to obtain the full θ_∞_(*r*_*c*_) dependence for that system. For example, Figure 3 (f) shows θ_∞_(*r*_*c*_) for ϵ_CO_ = 0.4247 kJ/mol (solid lines) extrapolated from θ_∞_(*r*_*c*_ = 3 nm), compared to θ_∞_(*r*_*c*_ = 1 nm) and θ_∞_(*r*_*c*_ = 2 nm) (from Figure 3 (b, c)), for graphene and graphite. These extrapolations of eq 4 from *r*_*c*_ = 3 nm exhibit excellent agreement with the simulation data for shorter cutoffs for all systems.

For graphene (blue line, Figure 3 (f)), the potential converges in the infinite cutoff limit to
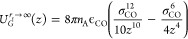
5where *n*_*A*_ is the areal number density of carbon atoms. For graphite (gray line, Figure 3 (f)), the potential in the infinite cutoff limit consists of a sum over terms identical to the RHS of eq 5, one for each sheet, which leads to a greater correction for graphite compared to graphene, as is apparent in Figure 3 (f).

By applying the correction in eq 4 to all points in Figure 3 (d), we obtain the results shown in Figure 3 (g). Fits of eq 1 to the data are plotted as solid lines. The experimental value of 60° for graphite (indicated by the lower dotted black line) serves as a reference to determine the corresponding  = 0.3807 kJ/mol for an infinite force cutoff (vertical dashed black line). Assuming the pairwise additivity of carbon–water interactions, the value of the graphene fit at  determines the contact angle for a free-standing graphene sheet, giving 80° (upper dotted black line). This value is within the error of the only experimental measurement available for a sessile droplet on (almost) free-standing graphene^31^ of 85° ± 5°, as well as the mean of the (albeit highly varied) distribution of experimental values 74° ± 35°. This slight hydrophilicity also agrees with the experimental fact that water spontaneously enters carbon nanotubes and planar graphene confinement.^78,79^ Thus, the MD simulation data in the *r*_*c*_ → ∞ limit form a bridge from the more reliable experimental contact angle of freshly exfoliated graphite to that of free-standing graphene.

To highlight the importance of the *r*_*c*_ → ∞ extrapolation, we circle back to Figure 3 (f), noting the significant θ_∞_(*r*_*c*_) dependence for graphite out to several nanometers. To map from the experimental graphite contact angle (where there is no LJ cutoff) to a ϵ_CO_ applicable to any graphenic surface/water system, θ_∞_(*r*_*c*_) must be obtained for large enough *r*_*c*_ that the dependence is insignificant. Otherwise, the resulting ϵ_CO_ will be too large and the calculated graphene contact angle too small.

We now have reference contact angles for both graphene and graphite and a single force field modeling both materials in the infinite cutoff limit. However, using very long cutoffs in MD simulations is computationally expensive. Therefore, we derive values of ϵ_CO_ for shorter cutoffs as well. Contact angles are plotted over ϵ_CO_ for a single sheet of graphene using a potential-shift scheme with *r*_*c*_ = 0.9 nm (in accordance with SPC/E) in Figure 3 (g) (orange × ’s), along with a fit of eq 1 (solid orange line). The ϵ_CO_ values where the fit gives the correct contact angles of graphene and graphite, respectively, are denoted by the orange dash-dotted lines at  = 0.4391 kJ/mol and  = 0.5164 kJ/mol. Taking these as force field parameters, surfaces with the wetting properties of graphene or graphite can be simulated using a single graphene sheet and SPC/E water. Although a *r*_*c*_ = 0.9 nm cutoff allows for interactions of water with two graphene sheets,  is given for a single graphene sheet to improve computational efficiency and simplicity. Quadratic fits that determine the lines in Figure 3 (a–d) are provided in the Supporting Information Section S5 for readers who wish to use different target contact angles or systems.

A similar procedure is carried out for several other popular water models. Values of ϵ_CO_ that reproduce the graphene and graphite contact angles of 80° and 60° on a single sheet of graphene, where σ_CO_ = 0.3367 nm and LJ forces are smoothly switched off between 1.0 and 1.2 nm, are presented in Table 2 for each water model. See the Supporting Information Section S9 for further details. These values are calculated using the planar simulation/pressure tensor method detailed below.

**Table 2:** ε_CO_ Values for Several Water Models That Reproduce Experimental Graphene– and Graphite–Water Contact Angles at a Single Sheet of Graphene with σ_CO_ = 0.3367 nm

water model	ϵ_CO,1.2_^graphene^ [kJ/mol]	ϵ_CO,1.2_^graphite^ [kJ/mol]
SPC/E^62^	0.406423	0.479049
TIP3P^80^	0.346613	0.415274
OPC3^81^	0.415578	0.497114
TIP4P-Ew^82^	0.412005	0.493531
TIP4P/2005^83^	0.434991	0.518667
OPC^84^	0.462782	0.548159
TIP5P-E^85^	0.393400	0.467202

Young’s equation,^86^ given by
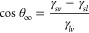
6relates the contact angle to the solid–vapor (γ_*sv*_), solid–liquid (γ_*sl*_), and liquid–vapor (γ_*lv*_) interfacial tensions. The interfacial tension for an entire system can easily be obtained from planar simulations using the pressure tensor via
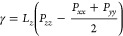
7where *L*_*z*_ is the system height and *P*_*αα*_ , α = *x*, *y*, *z*, are the diagonal elements of the pressure tensor.^86,87^Equation 7 can be thought of as giving the areal work needed for an infinitesimal, volume-preserving deformation where the system contracts along *z* and expands in the *xy*-plane.

The principal idea is to simulate three different flat planar systems as illustrated in Figure 4 (a–c) and use eq 7 to obtain the total system interfacial tensions, which can be written as
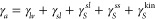
8
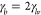
9
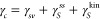
10where the liquid–vapor and solid–liquid interfacial tensions, γ_*lv*_ and γ_*sl*_, both arise due to deformations of the liquid,  and  denote interfacial tensions due to deformations of the solid working against liquid–solid interactions and solid–solid interactions, respectively, and  arises from the kinetic motion of the atoms of the solid. Here, it is assumed that the configuration of the solid surface in the system in Figure 4 (a) is not significantly changed by the presence of the liquid, such that the contributions  and  are the same as those for the system in Figure 4 (c). Substituting these into Young’s equation gives
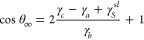
11which leaves only  unknown. See the Supporting Information Section S10 for a more complete discussion and derivation.

**Figure 4:**
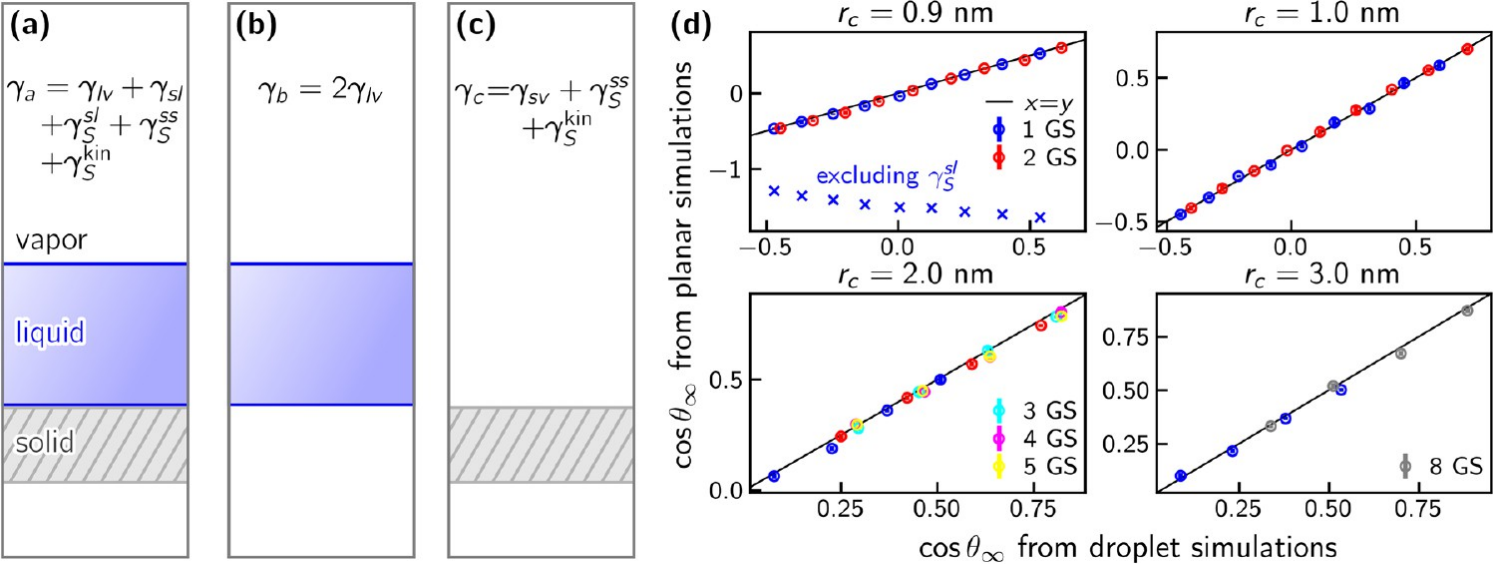
(a–c) Schematics of planar simulations to determine the system interfacial tensions, which are given in the figure. Contact angles can be calculated from these interfacial tensions. (d) Cosine of the macroscopic contact angle determined from planar simulations using eq 11 plotted over those from droplet simulations. Different panels show data for different LJ cutoffs. Solid black lines correspond to exact agreement. For comparison, results from planar simulations with  excluded are shown for a single graphene sheet and a cutoff of 0.9 nm (blue ×’s in the upper left panel).

From a physical perspective,  and γ_*c*_ appear in eq 11 because the deformation of the solid from which they arise is not involved in the spreading of a liquid droplet on a surface, and so must be subtracted out when calculating cos θ_∞_. The changes in the self-interaction and kinetic energy of the solid caused by the deformation are taken into account by subtracting γ_*c*_, while (perhaps less obviously) the corresponding change in the solid–liquid interaction energy is taken into account by subtracting . For single-component, monatomic, planar liquid and solid phases with atomic number densities *n*_*l*_(*z*) and *n*_*s*_(*z*) in a periodic box of dimensions *L*_*x*_ × *L*_*y*_ × *L*_*z*_, this term can be calculated directly as
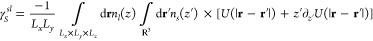
12where *U*(*r*) is the distance-dependent potential between interacting liquid and solid atoms a distance *r* apart.^88^ A detailed derivation and explanation can be found in the Supporting Information Section S10.

In Figure 4 (d), the values for cos θ_∞_ obtained from eq 11 (*y*-axis) are compared to the contact angles obtained from droplet simulations (*x*-axis) for all systems considered in this work. Regardless of the cutoff, number of graphene sheets, or ϵ_CO_, there is good agreement between the values across the entire range of cos θ_∞_.

Failing to include  leads to incorrect results, e.g., it consistently yields cos θ_∞_ < −1 for all systems investigated in this work, indicating complete dewetting (see upper left panel of Figure 4 (d), blue × ’s). This contradicts the findings of Sedlmeier et al.^39^ and Sendner et al.^40^ who find contact angles from droplet simulations and the planar pressure tensor approach to agree without accounting for . Their approach also differs slightly in that they use the virial tensor instead of the total pressure tensor, but as shown in the Supporting Information Section S11, we cannot reproduce the agreement they report without accounting for .

In this study, the water wetting behavior of graphene and graphite is explored in a systematic investigation of experimental graphene–/graphite–water contact angles in the literature. Graphene contact angles are found to vary extremely from study to study, an important message that serves to highlight the difficulty in their accurate measurement. Conversely, contact angles for graphite vary much less, with freshly exfoliated graphite exhibiting greater hydrophilicity, indicating that the hydrophobicity of not-freshly exfoliated graphite is due largely to contamination of the surface over time. Despite this, most force fields in the literature still target hydrophobic values for the contact angle of graphite.

To determine the contact angle of graphene, as well as consistent force fields for both graphene and graphite, we simulate droplets and measure contact angles geometrically using fits of liquid density profiles. We place significant emphasis on the dependence of the contact angle on the LJ cutoff, and extrapolate to the infinite cutoff limit, from which the optimal carbon–oxygen interaction strength, ϵ_CO_ = 0.3807 kJ/mol, needed to accurately reproduce the contact angle for graphite, is identified. The contact angle for free-standing graphene using this optimal ϵ_CO_ value is determined to be 80°, which is in good agreement with the available experimental data. We also determine the LJ interaction energies needed to reproduce these contact angles for shorter LJ cutoffs on a single sheet of graphene.

Additionally, we introduce a method that is ideally suited to graphenic surfaces for determining the contact angle from planar simulations using pressure tensors and the surface–liquid interaction potential. By accounting for the change to the surface–liquid interaction energy due to the surface’s deformation, which does not contribute to wetting phenomena, we are able to calculate contact angles correctly from only three planar simulations over the entire range of investigated values of ϵ_CO_ and cutoffs. While our method is ideal for graphite and graphene contact angles, which interact only via LJ potentials and are well described as continuum layers, these are not necessary conditions and the method is generalizeable to other surface types.

## Supporting Information Available

Experimental contact angles; molecular dynamics simulation details; one-dimensional droplet contact angle method; Lennard-Jones potential; fits of contact angle vs interaction strength; Lennard-Jones PME; surface tension of pure water; calculating free energy differences; graphene force fields for several water models; contact angles from pressure tensors; pressure tensor vs virial (PDF)
