# Supplementary material for: Modeling Water Interactions with Graphene and Graphite via Force Fields Consistent with Experimental Contact Angles
Source: J Phys Chem Lett. 2024 Jun 10;15(24):6325–33. doi: 10.1021/acs.jpclett.4c01143 (PMC11194815; doi:10.1021/acs.jpclett.4c01143)
Supplement: Supplementary file 1 — jz4c01143_si_001.pdf [file jz4c01143_si_001.pdf]

Supporting Information: Modeling Water Interactions with Graphene and Graphite via Force Fields Consistent with Experimental Contact Angles

Shane R. Carlson,[†,¶] Otto Schullian,[†,‡,¶] Maximilian R. Becker,[†] and Roland R. Netz[*,†]

†*Fachbereich Physik, Freie Universität Berlin, Arnimallee 14, D-14195 Berlin, Germany*

‡*Department of Biomaterials, Max Planck Institute of Colloids and Interfaces, 14424 Potsdam, Germany*

¶*Shane R. Carlson and Otto Schullian contributed equally to this work.*

E-mail: rnetz@physik.fu-berlin.de

S1 Experimental Contact Angles

An exhaustive literature review is performed to compare and analyze experimentally measured contact angles for graphene and graphite. The values for graphene are shown in Table S1 and can be separated into sessile-droplet and captive-bubble measurements. In the former, a droplet is deposited onto a graphene sheet (GS), whereas in the latter, a GS is floated on water while a small bubble is trapped underneath. Due to the thinness of the GS, the GS is deformed by the bubble which must be taken into account in the analysis. The captive bubble contact angle measurements have an average of $41.5° \pm 9.8°$. The sessile droplet contact angles vary widely from experiment to experiment, presumably due to the underlying supporting material and/or due to contamination, and have an average given by $74° \pm 35°$.

The experimental contact angles for graphite depend on the preparation of the graphite, specifically on whether it is freshly exfoliated (Table S2) or not (Table S3). Freshly exfoliated graphite constitutes a much more hydrophilic surface with an average contact angle of $60° \pm 13°$, whereas contact angles for not-freshly exfoliated graphite average around $89° \pm 10°$.

Table S1: Experimentally measured contact angles for graphene

ref.	year	method	contact angle
1	2008	reduction of GO on quartz	67.75°
2	2009	reduction of GO	127.0(4.0)°
3	2010	on SIC, chemically annealed	$\approx$ 92.5°
4	2011	CVD grown on Cu and transferred onto glass	93.8°
5	2012	CVD graphene on Si	33.2°
5	2012	CVD graphene on Au	78.8°
5	2012	CVD graphene on copper	86.2°
5	2012	CVD graphene on glass	48.1°
6	2012	CVD graphene on hydrophobic SiO_2	95°
6	2012	CVD graphene on hydrophilic SiO_2	40°
7	2013	GO heated to 700° C	143.2°
8	2013	graphene grown on Si-C material, annealed	72.9° $\pm$ 1.27°
9	2013	CVD graphene on copper	44°
10	2013	CVD grown on Copper, SiO_2, Glass, advancing	90.6°
11	2013	graphene from oxide	120°
12	2016	CVD grown on Cu and tranferred on glass	85° $\pm$ 5°
13	2018	CVD grown, on ice ($T = 0°$ C)	$\approx$ 30° $\pm$ 5°
13	2018	CVD grown, on agarose hydrogel	$\approx$ 10° $\pm$ 2°
14	2018	sessile droplet, on PMMA	54° $\pm$ 3°
14	2018	captive bubble, graphene floating on water	42° $\pm$ 7°
14	2018	captive bubble, at 50 % humidity on other side	42° $\pm$ 7°
14	2018	captive bubble, at 98 % humidity on other side	29° $\pm$ 8°
14	2018	captive bubble, on PMMA	53° $\pm$ 3°

Table S2: Experimentally measured contact angles for freshly exfoliated graphite

ref.	year	method	contact angle
15	1970	high-temperature/pressure annealed, freshly cleaved with tape, measurement using meniscus by immersion of plate	84.2°
16	1975	freshly cleaved using tape in air	63°
16	1975	freshly cleaved using tape in air, baked and evacuated at ultrahigh vacuum	35°
17	1980	freshly cleaved and vacuum heated graphite	42° ± 7°
3	2010	sessile droplet on freshly-cleaved HOPG	91.0°
18	2013	freshly cleaved using tape	62°
9	2013	freshly cleaved	64.4°
19	2014	cleaving in water and measurement in vapor	58°
19	2014	cleaving in air in clean room	53°
19	2014	cleaving in argon	45°
20	2014	freshly cleaved using tape	64.4° ± 2.9°
21	2015	freshly cleaved using tape, 3 different grades of graphite	56°
12	2016	freshly cleaved using tape, advancing	62.4° ± 0.9°
12	2016	freshly cleaved using tape, receding	60.2° ± 1.1°
13	2018	sessile droplet on freshly exfoliated graphite	61° ± 3°
14	2018	freshly cleaved using tape, sessile droplet	59° ± 3°
14	2018	freshly cleaved using tape, captive droplet	60° ± 3°

Table S3: Experimentally measured contact angles for not-freshly exfoliated graphite or where the production method is not mentioned

ref.	year	method	contact angle
22	1940	polished, washed with ethyl alcohol and dried, measured by immersion	85.6°
23	1997	no information	86°
2	2009	sessile droplet, static, graphite as provided	98.3°
4	2011	CVD grown on Ni and tranferred on glass	90.4°
5	2012	≥ 6 layers on copper, cvd grown and transferred	90.6°
5	2012	≥ 6 layers on glass, cvd grown and transferred	94.2°
18	2013	as received	95°
8	2013	grown (multilayers, 9-10 layers), annealed	91.6° ± 3.05°
9	2013	sessile droplet cleaved but left in air	91°
10	2013	sessile droplet	90.6°
19	2014	as received	91° ± 6°
20	2014	as it arrived from supplier	92.8° ± 2.7°
20	2014	cleaved and left in air for 7 days	97.0° ± 1.8°
21	2015	3 days old, cleaved using tape, 3 different grades of graphite	90°
12	2016	cleaved using tape, advancing, after one night	90° ± 1°
12	2016	cleaved using tape, receding, after one night	51.2° ± 1.5°

S2 Molecular Dynamics Simulation Details

Molecular Dynamics (MD) simulations are performed using GROMACS 2022[24,25] with the velocity-verlet integrator[26] and a timestep of 2 fs. The systems are simulated using periodic boundary conditions and the temperature is controlled using a velocity rescaling thermostat with a stochastic term[27] at 300 K. Water is modeled using the SPC/E water model, except where noted otherwise.[28] The non-polarizable carbons are frozen in hexagonal sheets with a bond length of 0.142 nm[29] and an inter-sheet distance of 0.34 nm.[30] The interaction between the graphene sheets and water consists solely of a Lennard-Jones (LJ) interaction between carbon and water oxygen, where the LJ length $\sigma_{\mathrm{CO}} = 0.3367$ nm is taken from the GROMOS53a6 forcefield.[31] The LJ strength ϵ_{CO} is varied over a range of 0.2580–0.5247 kJ/mol. Long-range electrostatic interactions are modeled using particle-mesh Ewald (PME) summation.[32] The Lennard-Jones force cutoff is varied between 0.9–3 nm. Only for the shortest cutoff of 0.9 nm is a potential shift used. For all other cutoffs, a force-switching scheme is used starting at a distance 0.1 nm shorter than the reported cutoff.

The systems simulated can be separated into two different groups: (i) systems that have a planar geometry, and (ii) droplet simulations. Planar geometry simulations consist either of a pure water slab with vapor above and below, or a surface comprising graphene sheets (GS) with the uppermost surface uniformly covered with a water slab. In either case, the surface and liquid slab extend over the periodic boundaries in the xy-plane. The simulation boxes are made nearly square in the xy-plane (they cannot be perfectly square as they must accommodate the different periodicities of the graphene in the x and y directions) and much longer in the z direction. For the droplet simulations, the periodic box is made short in the x-direction and long in y direction to allow the formation of cylindrical droplets extending over the periodic boundary along the x-direction. The amount of water is chosen such that there is enough water to form a droplet but not so much that it tends to spontaneously form a slab covering the entire GS. The box sizes and particle numbers are shown in Table S4. In addition, in all simulations, a flat-bottom potential in the z-direction, with a force constant of $k = 1000$ kJ/(mol nm^2), is used to avoid water attaching on the lower side of the GS. The potential is positioned in such a way that the region where no force is exerted includes both the water and the graphene sheet, and spans a width of 16–50 nm (depending on the overall system size). Any time a water molecule strays above the potential's flat bottom, a force acts to reflect it back down towards the GS.

Systems are equilibrated for 10 ns before production simulations begin. The length of the production simulation depends on the system size and is shown in Table S4. For data analysis, trajectories are cut into 10 ns-long subtrajectories to obtain the standard deviation.

Table S4: Simulation parameters for all systems in this work

(a) 0.9 nm potential-shift Lennard-Jones interactions

system	water mol.	carbons	L_x (nm)	L_y (nm)	L_z (nm)	prod. (ns)
pure water	2165	0	4.05	4.05	16.00	1000
planar system	560	216–432	2.56	2.21	25.00	100
droplet	550–3453	2016–4032	2.56	20.66	25.00	100

(b) 0.9–1.0 nm force-switch Lennard-Jones interactions

system	water mol.	carbons	L_x (nm)	L_y (nm)	L_z (nm)	prod. (ns)
pure water	500	0	2.56	2.95	60.00	1000
planar system	1904	288–576	2.56	2.95	60.00	200
droplet	1183–8842	2952–5904	2.56	30.25	60.00	200

(c) 1.9–2.0 nm force-switch Lennard-Jones interactions

system	water mol.	carbons	L_x (nm)	L_y (nm)	L_z (nm)	prod. (ns)
pure water	2165	0	4.05	4.05	16.00	1000
planar system	2649	720–3600	4.26	4.43	20.00	100
droplet	1233–5679	4920–24600	4.26	30.25	20.00	100

(d) 2.9–3.0 nm force-switch Lennard-Jones interactions

system	water mol.	carbons	L_x (nm)	L_y (nm)	L_z (nm)	prod. (ns)
pure water	7029	0	6.05	6.05	20.00	282
planar system	8884	1728–13824	6.82	6.64	25.00	100
droplet	2595–10373	10560–84480	6.82	40.58	25.00	100

S3 One-Dimensional Droplet Contact Angle Method

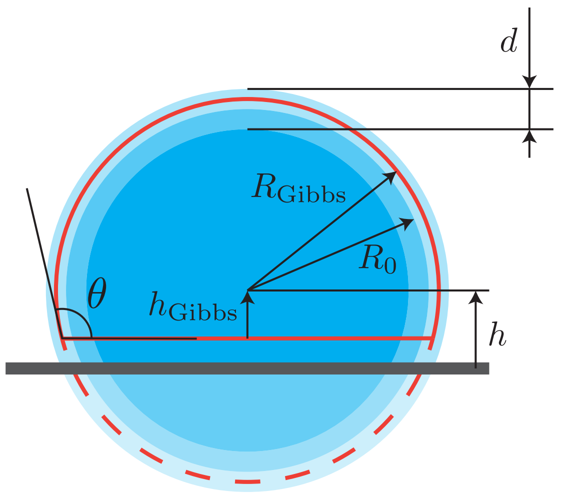

Figure S1: Schematic of a droplet on a solid surface illustrating some important geometrical definitions. Note the difference between the droplet radius measured to the Gibbs dividing surface R_{Gibbs}, and that extracted from the sigmoid function fit (see Eq. (S5)), R_0, as well as the difference between the droplet center height measured from the solid surface, h, and that measured from the liquid Gibbs dividing surface near the solid, h_{Gibbs}. The contact angle, θ, is taken as the angle between the two Gibbs dividing surfaces (red lines). The sigmoid width d is also shown.

A contact angle θ can be defined for a droplet adsorbed on a flat surface; it is generally taken to be the inside angle formed where the side of the droplet meets the surface, as illustrated in Fig. S1. The contact angle can be determined from the droplet height and radius. In order to determine the cylindrical droplet geometry, the aim is to fit the density to

$$\rho(y,z) = \frac{\rho_{\mathrm{planar}}(z)}{2}\left(1 - \tanh\left(\frac{\sqrt{y^2 + (z-h)^2} - R_0}{d}\right)\right), \tag{S1}$$

where $\rho_{\mathrm{planar}}(z)$ is the density profile of a planar system, i.e., the density of a water slab that fully covers the GS. $\rho_{\mathrm{planar}}(z)$ is determined from a separate simulation and an example is shown in Fig. S2 (a). The carbons of the GS that are closest to the water are taken to be at $z = 0$. The water density sharply increases and features peaks that decay to the bulk density of water within 15 Å of the interface. The bulk density is calculated from the liquid phase 20 Å above the highest graphene layer and $\rho_{\mathrm{planar}}(z)$ is set to this bulk value for all $z \geq 20$ Å, as is shown in Fig. S2 (a).

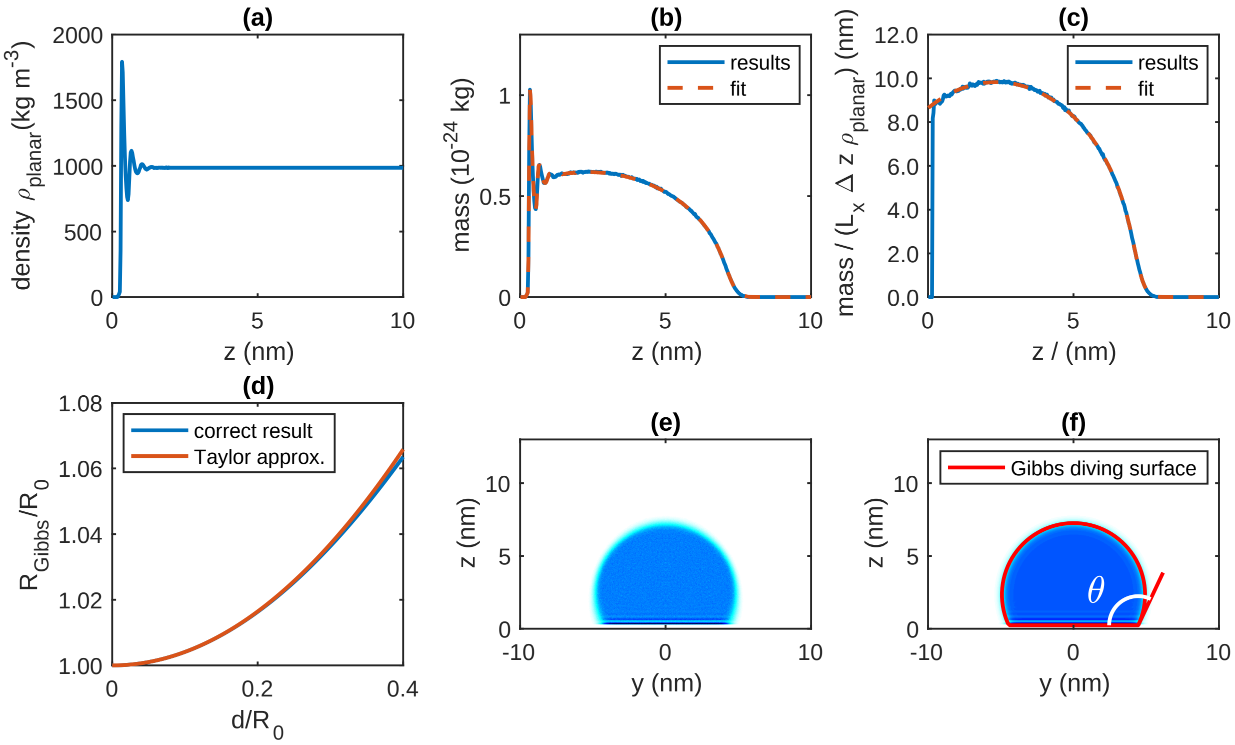

Figure S2: **(a)** Density profile of a planar water slab on a GS as a function of distance to the graphene. This profile is set to the bulk density value for all $z \geq 20$ Å. **(b)** Density profile of a droplet as a function of distance to the graphene, determined from an MD simulation (blue line). Here, the density is taken to be the total mass per slab of thickness $\Delta z = 0.25$ nm. The red dashed line is calculated from the fit parameters obtained in (c). **(c)** Corrected density profile (Eq. (S2) and (S3)), i.e., the density profile divided by the planar density (a), and fit of Eq. (S4) to determine the droplet shape determined by the parameters d, h, and R_0. **(d)** The rescaled Gibbs radius R_{Gibbs}/R_0 of a free cylindrical droplet as a function of the ratio of fit parameters d/R_0 (Eq. (S5)) compared to the Taylor expansion (Eq. (S6)). **(e)** Two-dimensional density of a cylindrical droplet from an MD simulation. **(f)** The two-dimensional density (Eq. (S1)) reconstructed using the parameters d, h, and R_0 from the fit (b). The red line is the Gibbs dividing surface and the contact angle of the droplet is denoted as θ.

To avoid a two-dimensional fit, we integrate the density in Eq. (S1) to obtain the mass in a slab of thickness $\Delta z = 0.25$ nm, parallel to the graphene layer, as a function of the height

$$m(z) = \frac{L_x \Delta z \rho_{\text{planar}}(z)}{2} \int_{-L_y/2}^{L_y/2} \left(1 - \tanh\left(\frac{\sqrt{y^2 + (z-h)^2} - R_0}{d}\right)\right) \mathrm{d}y \,. \tag{S2}$$

The numerical fit is then performed for

$$\frac{m(z)}{L_x \Delta z \rho_{\text{planar}}(z)} \tag{S3}$$

using the numerically evaluated integral

$$\int_{-L_y/2}^{L_y/2} \left(1 - \tanh\left(\frac{\sqrt{y^2 + (z-h)^2} - R_0}{d}\right)\right) \mathrm{d}y \tag{S4}$$

to determine h, R_0 and d for $z > 3$ Å. The result of the fit is shown in Fig. S2 (c). Equivalently, the mass given in Eq. (S2) using the obtained fitting parameters is compared to the simulation results in Fig. S2 (b). Fig. S2 (e) shows the 2D-density of the droplet determined from simulation and Fig. S2 (f) shows the density calculated using Eq. (S1) with the parameters determined in Fig. S2 (c). The advantage of the method is that the fit completely describes the density of the droplet, including the high-density peaks close to the interface and the decay at the upper boundary of the droplet. In addition, for the fit itself only a one-dimensional binning and no centering is necessary, which makes the procedure much faster than the two-dimensional binning and fitting procedures used in current literature[6,30,33,34] (see below).

Here, we take the contact angle to be defined as the angle at the crossing of the Gibbs dividing surface of the liquid-vapor interface and the Gibbs dividing surface of the solid-liquid interface, as illustrated in Fig. S1. The Gibbs dividing surface is defined as the surface, which encloses a phase, such that if the enclosed space were filled with the constant bulk density, it would contain the same amount of particles. In the case of a planar interface whose density is described by a sigmoid $\propto 1 - \tanh((z - R_0)/d)$, the Gibbs dividing surface lies at height R_0 and is identical to the surface where $\tanh((z - R_0)/d) = 0$, i.e. where the density is half the bulk value. This is, however, not true for a curved interface such as a cylindrical droplet.

In our case, to determine the contact angle, we need to determine the Gibbs dividing surfaces of a cylindrical droplet, characterized by a perpendicular cross-section resembling a circular segment. A circular segment can be described by the radius R_{Gibbs} and the height h_{Gibbs}, which represents the distance from where the disk is "cut" by the planar surface to the center of the disk, i.e., $-R_{\mathrm{Gibbs}} \leq h_{\mathrm{Gibbs}} \leq R_{\mathrm{Gibbs}}$. To determine the parameters R_{Gibbs} and h_{Gibbs} from the fitting parameters R_0, d and h we need to identify first the location of the curved (vapor-liquid) interface and, subsequently, the planar (solid-liquid) interface. In other words, we focus initially on determining R_{Gibbs} by examining a cylindrical droplet that is not attached to a surface. The density as a function of the distance to the droplet center (i.e., the axis of the cylinder) is approximated by

$$\rho_{\mathrm{cyl}}(r) = \frac{\rho_{\mathrm{bulk}}}{2}\left(1 - \tanh\left(\frac{r - R_0}{d}\right)\right) .$$

The total mass of the liquid in a given cylindrical droplet of height L_x is given by

$$\begin{aligned} m_{\text{tot,cyl}} &= L_x 2\pi \int_0^\infty r\rho(r)\mathrm{d}r \\ &= -\frac{1}{2} L_x d^2 \pi \rho_{\text{bulk}} \mathrm{Li}_2(e^{-2R_0/d}))\,, \end{aligned}$$

where $\mathrm{Li}_2(\cdot)$ is the Polylogarithm Function. Following the definition of the Gibbs dividing surface, the radius is related to the total mass by

$$m_{\text{tot,cyl}} = L_x \pi R_{\text{Gibbs}}^2 \rho_{\text{bulk}}\,,$$

and hence

$$\frac{R_{\text{Gibbs}}}{R_0} = \frac{d}{R_0}\sqrt{-\frac{\mathrm{Li}_2(e^{-2R_0/d}))}{2}}\,. \tag{S5}$$

The ratio of the Gibbs-dividing-surface radius to the radius R_0 for a cylindrical droplet without a surface only depends on the ratio of d/R_0 and can be seen in Fig. S2 (d). For thin boundary layers ($d/R_0 \ll 1$) the ratio R_{Gibbs}/R_0 is almost 1. Up to $d/R_0 \approx 0.2$ it can be approximated very well with a Taylor expansion

$$\frac{R_{\text{Gibbs}}}{R_0} \approx 1 + \frac{\pi^2}{24}\left(\frac{d}{R_0}\right)^2\,, \tag{S6}$$

as seen in Fig. S2 (d). To determine h_{Gibbs}, i.e., the distance between where the cylindrical droplet is "cut" and where it is positioned on the GS, we use a cylindrical cap that is cut at the distance h_{Gibbs} from the cylinder axis,

$$\begin{aligned} n_{H_2O} \cdot m_{H_2O} &= m_{\text{droplet}} \\ &= \rho_{\text{bulk}} L_x \frac{1}{2}\Bigg[-2h_{\text{Gibbs}}\sqrt{R_{\text{Gibbs}}^2 - h_{\text{Gibbs}}^2} \\ &\quad -2R_{\text{Gibbs}}^2 \tan^{-1}\left(\frac{h_{\text{Gibbs}}}{\sqrt{R_{\text{Gibbs}}^2 - h_{\text{Gibbs}}^2}}\right) + \pi R_{\text{Gibbs}}^2\Bigg]\,, \end{aligned} \tag{S7}$$

where the terms inside the brackets correspond to the area of a circular segment. The value for R_{Gibbs} is calculated using Eq. (S6), and h_{Gibbs} is found by numerically solving Eq. (S7). Finally, the contact angle can be calculated as $\theta = \cos^{-1}(h_{\text{Gibbs}}/R_{\text{Gibbs}})$. The Gibbs dividing surfaces and contact angle for the profile in Fig. S2 (b) are shown in Fig. S2 (f) along with the calculated 2D-density.

Performance: The time needed to generate the density histograms needed for the 1D method presented above is compared in Fig. S3 to that for generating 2D histograms used for calculating contact angles from a previous publication.[34] Specifically, mean wall time per frame is measured. Here, wall time refers to the total time needed for a computer to perform a computation. The systems tested consist of cylindrical droplets on graphene of 1024, 2048, 4096, and 8192 water molecules (N_{water}). In both methods, the system is binned along the surface normal, z, with a bin width of 0.25 Å. For the 2D method, the radial direction is also binned with the same width.

Least squares fits are also shown in Fig. S3 and both methods appear to give roughly power law scaling in N_{water}, with the 2D method scaling close to $\propto N_{\text{water}}^2$, and the 1D method scaling slower than $\propto N_{\text{water}}^1$. For the systems studied, the speed up to calculate 1D histograms varied from about 30× for the smallest system, to over 400× for the largest.

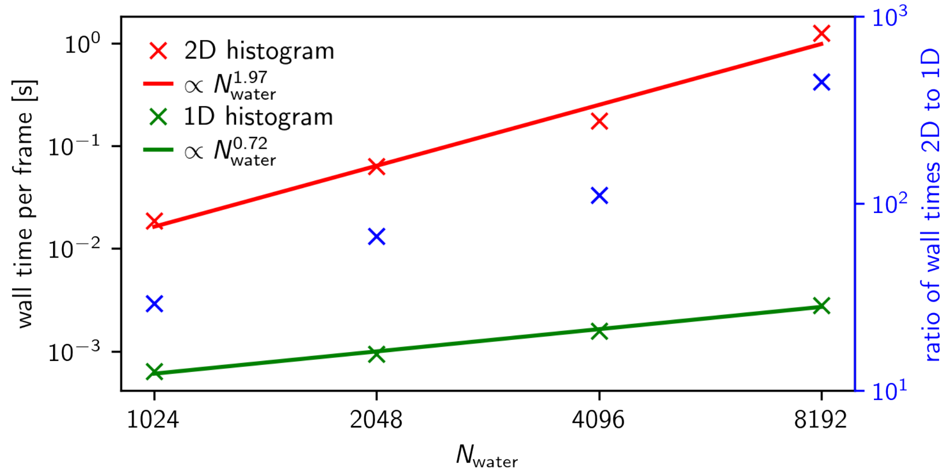

Figure S3: Time per frame to calculate mass density histograms of cylindrical droplets needed for the 1D-histrogram and 2D-histogram methods, as a function of the number of water molecules. Bins are 0.25 Å wide for both methods. Fits show the approximate power law scaling behavior over the number of molecules. The right axis (blue data) shows the ratio of times per frame between the two methods, which illustrates the speed advantage of the 1D-histogram method.

S4 Lennard-Jones Potential

The Lennard-Jones (LJ) interaction is modeled using the 12-6 potential given by

$$U_{12-6}(r) = 4\epsilon \left(\frac{\sigma^{12}}{r^{12}} - \frac{\sigma^6}{r^6} \right) , \tag{S8}$$

where r is the distance between the two interacting particles. In simulations, the long-range contribution of this interaction is set to zero in one of two ways. One option is a potential shift, where the potential is set to zero beyond a cutoff r_c and shifted upward such that it is continuous,

$$U_{\mathrm{LJ}}(r, r_c) = \begin{cases} U_{12-6}(r) - U_{12-6}(r_c) & \text{for } 0 < r \leq r_c \\ 0 & \text{otherwise} \end{cases} . \tag{S9}$$

The potential-shift scheme has the disadvantage that the force is discontinuous at $r = r_c$. Alternatively, a force-switching scheme can be used, where two cutoffs r_s and r_c are required. Here, the force decays linearly to zero between r_s and r_c, namely

$$U_{\mathrm{LJ}}(r, r_s, r_c) = \begin{cases} U_{12-6}(r) - U_{12-6}(r_s) - b(r_s - r_c)^2 & \text{for } 0 < r \leq r_s \\ -b(r - r_c)^2 & \text{for } r_s < r \leq r_c \\ 0 & \text{otherwise} \end{cases} ,$$

where b is a constant, determined by the condition that the force is continuous, which yields

$$-4\epsilon_{CO} \left(-12\frac{\sigma^{12}}{r_s^{13}} + 6\frac{\sigma^6}{r_s^7} \right) = 2b(r_s - r_c) \qquad \Rightarrow \qquad b = \frac{2\epsilon_{CO}}{(r_s - r_c)} \left(12\frac{\sigma^{12}}{r_s^{13}} - 6\frac{\sigma^6}{r_s^7} \right) .$$

We approximate the carbons in a graphene sheet as a continuum with unifom areal number density n_A spanning the xy-plane at height z_0. The total LJ potential at height z is

$$U_G(z, z_0) = n_A \int_{\mathbb{R}^2} U_{LJ} \left(\sqrt{x^2 + y^2 + (z - z_0)^2}, r_s, r_c \right) \mathrm{d}x\mathrm{d}y \, . \tag{S10}$$

Eq. (S10) can be solved analytically for the force-switch and potential-shift schemes. However, we solve this equation numerically. In the case of an infinite cutoff, where the LJ interaction converges to the 12-6 potential, it is given by

$$U_G(z, z_0) = 8\pi n_A \epsilon \left(\frac{\sigma^{12}}{10(z - z_0)^{10}} - \frac{\sigma^6}{4(z - z_0)^4} \right) , \tag{S11}$$

which is reproduced in the main text as Eq. (5).

S5 Fits of Contact Angle vs. Interaction Strength

In order to provide the contact angle for the entire range of investigated values for ϵ_{CO} we fit the results shown in Fig. 3 (a–d) in the main text with

$$\cos\theta_\infty = a\epsilon_{\mathrm{CO}}^2 + b\epsilon_{\mathrm{CO}} + c\,. \tag{S12}$$

For example, the fit is shown for the droplet simulations using the cutoff of $r_c = 0.9$ nm for one and two GS in Fig. S4. The resulting parameters for all systems and parameters considered are given in Table S5. Note that long-range electrostatics are handled using PME for all systems.

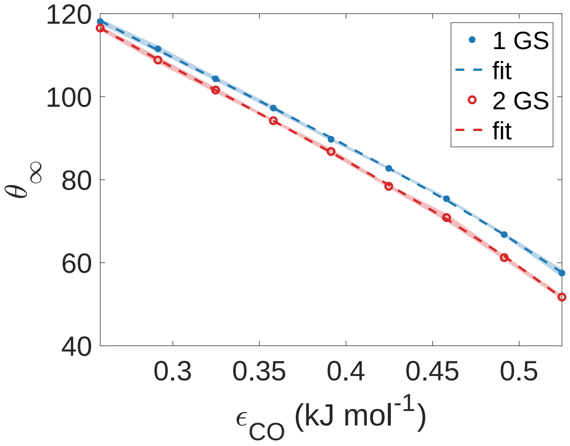

Figure S4: The contact angle as a function of ϵ_{CO} for a cutoff of $r_c = 0.9$ nm and potential shift scheme for a single graphene sheet (blue) and two sheets (red). The dashed lines are least-squares fits of Eq. (S12). The shaded area corresponds to the standard error of the mean, which is difficult to see because it is close to the same thickness as the dashed line.

Table S5: Parameters for quadratic fits in Fig. 3 (a–d) in the main text

(a) 0.9 nm potential-shift Lennard-Jones interactions

no. of graphene layers	a [kJ^{-2} mol^2]	b [kJ^{-1} mol]	c
1	1.858	2.329	-1.199
2	1.598	2.754	-1.263

(b) 0.9–1.0 nm force-switch Lennard-Jones interactions

no. of graphene layers	a [kJ^{-2} mol^2]	b [kJ^{-1} mol]	c
1	1.778	2.513	-1.213
2	1.896	2.667	-1.217

(c) 1.9–2.0 nm force-switch Lennard-Jones interactions

no. of graphene layers	a [kJ^{-2} mol^2]	b [kJ^{-1} mol]	c
1	-2.488	6.339	-1.874
2	1.604	3.858	-1.337
3	4.620	1.375	-0.790
4	1.602	3.981	-1.341
5	3.174	2.704	-1.084

(d) 2.9–3.0 nm force-switch Lennard-Jones interactions

no. of graphene layers	a [kJ^{-2} mol^2]	b [kJ^{-1} mol]	c
1	2.331	2.560	-1.128
8	2.886	3.123	-1.150

S6 Lennard-Jones PME

GROMACS offers the option to model long-range LJ interactions using a particle-mesh-Ewald (PME) scheme, which should theoretically correspond to an infinite force cutoff.[35] Similarly to the electrostatic PME, interactions are divided into long-range and short-range components. Short-range interactions are calculated in the standard fashion using Eq. (S8), while long-range interactions are calculated in Fourier space using the LJPME routine.

We carefully studied the long-range forces and potential energies produced by the LJPME routine in GROMACS 2022.0, and found the algorithm to give inaccurate forces and potential energies for typical values of `fourierspacing`, `ewald-rtol-lj`, and `rvdw`.

Fig. S5 (a) shows the force (output by GROMACS) between two argon atoms (no electric charge; LJ interactions only) in vacuum in a 10×10×20 nm box as a function of their separation distance d. Here, the dashed portions of the curves represent negative data (corresponding to a repulsive force). For each curve, the corresponding cutoff `rvdw`, beyond which forces are calculated in Fourier-space, is shown as a vertical dashed line of the same color as that curve. The black curve is for a cutoff of `rvdw` of 4 nm, and is shown as a reference, as all plotted data there is calculated in real space. In the left panel, `fourierspacing` is 0.12 nm, and in the right, 0.06 nm. For all data shown, `ewald-rtol-lj` is relaxed to 0.01, which gives better results than 0.001. For all cutoffs tested, the LJ force begins to show non-physical oscillatory behavior at some point beyond `rvdw`. This oscillatory behavior is somewhat mitigated by reducing `fourierspacing` and increasing `rvdw`, as is evident in Fig. S5 (a), as well as relaxing (i.e. increasing) `ewald-rtol-lj`. Thus, given the correct choice of parameters, the accuracy of the LJPME routine might be sufficient for a given application. However, we find accuracy to come at major computational expense, especially for large systems.

We carried out benchmarking simulations using single-precision GPU-enabled GROMACS 2022.0 on 8 cores of an AMD Ryzen Threadripper 3970X CPU with an Nvidia GeForce RTX 2080 GPU. Fig. S5 (b) shows wall times plotted over `rvdw` for NVT simulations of 4096 water molecules in bulk in a 4.697-nm cubic box (left panel) and in droplet form in a much larger 10-nm cubic box (right panel). Data are shown for a simple force-switching scheme (*not* LJPME), and for LJPME with `fourierspacing` of 0.12 and 0.06 nm and `ewald-rtol-lj` of 0.01. For the small system, using LJPME with `fourierspacing` of 0.06 nm results in a slowdown of roughly an order of magnitude compared to force-switching. For the large system, this slowdown approaches two orders of magnitude. For this reason, we think it prudent to circumvent the use of LJPME in many cases.

Curiously, the surface tension does not exhibit significantly inconsistent behavior when

calculated using LJPME. In the main text, we show that the surface tension scales with r_c^{-2}, where r_c is the finite-force cutoff. Fig. S5 (c) shows the surface tensions for water determined using finite force cutoffs in addition to the surface tension determined using LJPME with `fourierspacing` = 0.1 nm, `rvdw` = 1.0 nm, and `ewald-rtol-lj` = 0.001 (plotted at $r_c^{-2} = 0$ as LJPME should correspond physically to an infinite cutoff). Indeed the surface tension found using LJPME agrees, within error, to the linear fit (blue line) of the finite-cutoff data. This serves as a caution against the use of the surface tension alone as a test of long-range potentials.

The computational expense/inaccuracy dilemma of LJPME lead us to instead use an analytical extrapolation to infinite cutoff (see Fig. 3 (f) in the main text and the related discussion).

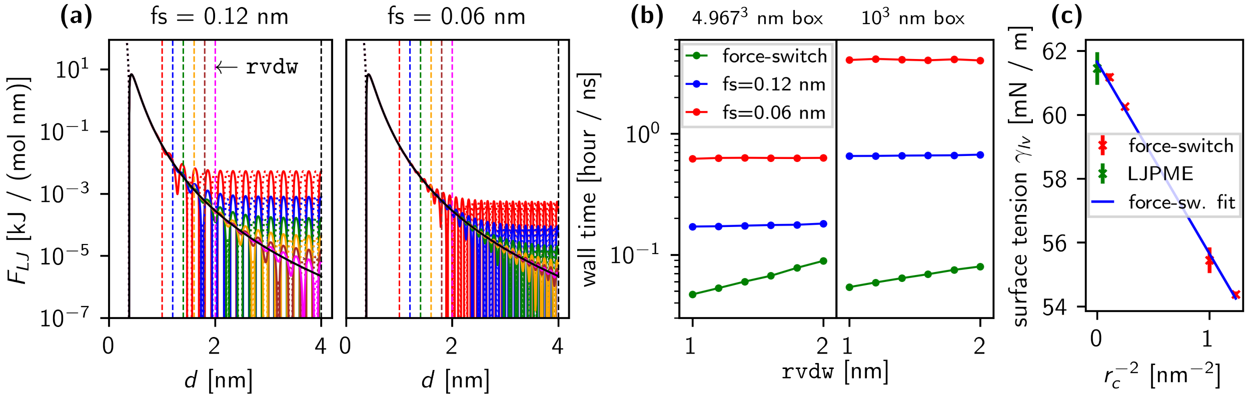

Figure S5: **(a)** Lennard-Jones force between two argon atoms in vacuum in a 10×10×20 nm box plotted over their separation distance d, illustrating the inaccuracy of the LJPME routine for some parameter regimes. The dashed portions of curves represent negative data, which indicates a repulsive force. Differently colored curves represent different cutoffs `rvdw`; the corresponding cutoff is shown as a vertical dashed line of matching color. The black curve, for `rvdw` = 4 nm, is shown as a reference. Left panel: `fourierspacing` = 0.12 nm; Right panel: `fourierspacing` = 0.06 nm. `ewald-rtol-lj` = 0.01 for all data shown. **(b)** Wall times for NVT simulations of 4096 water molecules plotted over `rvdw`, illustrating the high computational cost of LJPME for some parameter regimes. The left panel is for bulk water in a 4.697-nm cubic box, while the right is for a water droplet in a large 10-nm cubic box. Data are shown for a force-switching scheme and for LJPME with `fourierspacing` of 0.12 and 0.06 nm and `ewald-rtol-lj` of 0.01. **(c)** Surface tension γ_{lv} of water over the inverse square of the finite-force cutoff r_c, along with a linear fit. The LJPME result (`fourierspacing` = 0.1 nm, `rvdw` = 1.0 nm, `ewald-rtol-lj` = 0.001) is also shown at the infinite-cutoff limit, and agrees (presumably coincidentally) within error with the fit.

S7 Surface Tension of Pure Water

We determine the surface tension for pure water from a simulation of a planar water slab as illustrated in Fig. 4 (b) in the main text. The trajectories are divided into ten short trajectories of equal length to determine the standard error of the mean. The tension is calculated using the pressure tensor via

$$\gamma_{lv} = \frac{L_z}{2}\left(P_{zz} - \frac{P_{xx}+P_{yy}}{2}\right) , \tag{S13}$$

and the resulting values for different LJ cutoffs are given in Table S6.

Table S6: Liquid–vapor surface tension of SPC/E water for a range of cutoffs

cutoff r_c (nm)	scheme	surface tension γ_{lv} (mN m^{-1})
0.9	potential shift	54.379 ± 0.098
0.9–1	force switch	55.45 ± 0.40
1.9–2	force switch	60.26 ± 0.060
2.9–3	force switch	61.18 ± 0.12

S8 Calculating Free Energy Differences

The canonical partition function is given by

$$Z = \int \mathrm{d}\Gamma \, e^{-\beta \mathcal{H}(\Gamma)} \,, \tag{S14}$$

where Γ is the phase-space variable specifying the system microstate, $\int \mathrm{d}\Gamma$ denotes an integral over all phase space, $\beta = \frac{1}{k_B T}$ is the inverse thermal energy, and $\mathcal{H}$ is the Hamiltonian of the system. The Helmholz free energy is given by

$$\mathcal{F} = -k_B T \log Z \,. \tag{S15}$$

Let the Hamiltonian depend on a variable λ that characterizes the interactions in the system. Then the derivative of $\mathcal{F}$ with respect to λ is given by

$$\begin{aligned}
\frac{\partial \mathcal{F}}{\partial \lambda} &= -\frac{\partial}{\partial \lambda} k_B T \log Z(\lambda) \\
&= -\frac{k_B T}{Z(\lambda)} \frac{\partial Z(\lambda)}{\partial \lambda} \\
&= -\frac{k_B T}{Z(\lambda)} \frac{\partial}{\partial \lambda} \int \mathrm{d}\Gamma \, e^{-\beta \mathcal{H}(\Gamma,\lambda)} \\
&= \frac{1}{Z(\lambda)} \int \mathrm{d}\Gamma \, e^{-\beta \mathcal{H}(\Gamma,\lambda)} \frac{\partial \mathcal{H}(\Gamma,\lambda)}{\partial \lambda} \\
&= \left\langle \frac{\partial \mathcal{H}(\Gamma,\lambda)}{\partial \lambda} \right\rangle_\lambda \,,
\end{aligned} \tag{S16}$$

where the expectation value $\langle \ldots \rangle_\lambda$ is the expectation value for the ensemble with the Hamiltonian $\mathcal{H}(\Gamma, \lambda)$. Integrating both sides yields the thermodynamic integration equation,

$$\int_{\lambda_1}^{\lambda_2} \mathrm{d}\lambda \, \frac{\partial \mathcal{F}}{\partial \lambda} = \mathcal{F}_2 - \mathcal{F}_1 = \Delta \mathcal{F} = \int_{\lambda_1}^{\lambda_2} \mathrm{d}\lambda \, \left\langle \frac{\partial \mathcal{H}(\Gamma,\lambda)}{\partial \lambda} \right\rangle_\lambda \,, \tag{S17}$$

which can be used to calculate the free energy difference between two ensembles 1 and 2. Consider a system consisting of a solid surface and an adsorbed liquid phase. Let $\mathcal{H}^{sl}$ be the part of the Hamiltonian consisting of the potential energy of the solid-liquid interactions, and $\mathcal{H}^0$ be the rest, i.e., the Hamiltonian for the adsorbed state is $\mathcal{H}_a = \mathcal{H}^0 + \mathcal{H}^{sl}$. If the liquid is detached from the solid, the Hamiltonian is given simply by $\mathcal{H}_d = \mathcal{H}^0$. The work of adhesion W is the difference in free energy between states where the liquid is adsorbed on

the surface, $\mathcal{F}_a$, and where it is fully detached, $\mathcal{F}_d$,

$$\begin{aligned} W(\lambda) &= \mathcal{F}_d(\lambda) - \mathcal{F}_a(\lambda) \\ &= k_B T \log \frac{Z_a(\lambda)}{Z_d(\lambda)} \,, \end{aligned} \tag{S18}$$

where Z_a and Z_d are the canonical partition functions for the adsorbed and detached states, respectively. The derivative with respect to λ is given by

$$\frac{\partial W(\lambda)}{\partial \lambda} = k_B T \frac{Z_d(\lambda)}{Z_a(\lambda)} \frac{\partial}{\partial \lambda} \frac{Z_a(\lambda)}{Z_d(\lambda)} \,. \tag{S19}$$

We first consider the case where $\mathcal{H}^{sl} = \mathcal{H}^{sl}(\Gamma, \lambda)$ depends on λ, while $\mathcal{H}^0 = \mathcal{H}^0(\Gamma)$ does not. This is the case for $\lambda = \epsilon^{sl}$, the solid-liquid Lennard-Jones interaction strength, and where λ parameterizes the number of (spatially frozen) graphene sheets. Here, Z_d is independent of λ, and Eq. (S19) gives

$$\frac{\partial W(\lambda)}{\partial \lambda} = \frac{k_B T}{Z_a(\lambda)} \frac{\partial Z_a(\lambda)}{\partial \lambda} = \frac{k_B T}{Z_a(\lambda)} \int \mathrm{d}\Gamma \, e^{-\beta\left(\mathcal{H}^0 + \mathcal{H}^{sl}\right)} \left(-\beta \frac{\partial \mathcal{H}^{sl}}{\partial \lambda}\right) = - \left\langle \frac{\partial \mathcal{H}^{sl}}{\partial \lambda} \right\rangle_{\lambda, a} \,. \tag{S20}$$

Then, analogously to Eq. (S17), the difference in works of adhesion between ensembles 1 and 2 is given by

$$\Delta W = W(\lambda_2) - W(\lambda_1) = \int_{\lambda_1}^{\lambda_2} \mathrm{d}\lambda \, \frac{\partial W(\lambda)}{\partial \lambda} = - \int_{\lambda_1}^{\lambda_2} \mathrm{d}\lambda \, \left\langle \frac{\partial \mathcal{H}^{sl}}{\partial \lambda} \right\rangle_{\lambda, a} \,. \tag{S21}$$

Next, we consider the where case $\mathcal{H}^0 = \mathcal{H}^0(\Gamma, \lambda)$ also depends on λ. This is the case for $\lambda = r_c$, the Lennard-Jones force cutoff distance. Here, taking the derivative in Eq. (S19) yields the more complicated expression

$$\begin{aligned} \frac{\partial W(\lambda)}{\partial \lambda} &= \frac{\int \mathrm{d}\Gamma \, e^{-\beta \mathcal{H}^0} \frac{\partial \mathcal{H}^0}{\partial \lambda}}{\int \mathrm{d}\Gamma \, e^{-\beta \mathcal{H}^0}} - \frac{\int \mathrm{d}\Gamma \, e^{-\beta\left(\mathcal{H}^0 + \mathcal{H}^{sl}\right)} \frac{\partial \mathcal{H}^0}{\partial \lambda}}{\int \mathrm{d}\Gamma \, e^{-\beta\left(\mathcal{H}^0 + \mathcal{H}^{sl}\right)}} - \frac{\int \mathrm{d}\Gamma \, e^{-\beta\left(\mathcal{H}^0 + \mathcal{H}^{sl}\right)} \frac{\partial \mathcal{H}^{sl}}{\partial \lambda}}{\int \mathrm{d}\Gamma \, e^{-\beta\left(\mathcal{H}^0 + \mathcal{H}^{sl}\right)}} \\ &= \underbrace{\left\langle \frac{\partial \mathcal{H}^0}{\partial \lambda} \right\rangle_{\lambda, d} - \left\langle \frac{\partial \mathcal{H}^0}{\partial \lambda} \right\rangle_{\lambda, a}}_{\equiv \frac{\partial W^0}{\partial \lambda}} - \left\langle \frac{\partial \mathcal{H}^{sl}}{\partial \lambda} \right\rangle_{\lambda, a} \,. \end{aligned} \tag{S22}$$

We expect the two expectation values comprising $\partial W^0/\partial\lambda$ to be very similar, and accordingly $\partial W^0/\partial\lambda \approx 0$. This is because the liquid and solid configurations, taken individually, do not change significantly upon adsorption of the liquid, i.e., $\mathcal{H}^0$ will remain largely unchanged

between these two states. More concretely, from Eq. (S22), it follows that

$$\begin{aligned}\frac{\partial W^0(\lambda)}{\partial\lambda} &= \frac{\int \mathrm{d}\Gamma\, e^{-\beta\mathcal{H}^0}\frac{\partial\mathcal{H}^0}{\partial\lambda}}{\int \mathrm{d}\Gamma\, e^{-\beta\mathcal{H}^0}} - \left\langle\frac{\partial\mathcal{H}^0}{\partial\lambda}\right\rangle_{\lambda,a} \\ &= \frac{\int \mathrm{d}\Gamma\, e^{-\beta\left(\mathcal{H}^0+\mathcal{H}^{sl}\right)}e^{\beta\mathcal{H}^{sl}}\frac{\partial\mathcal{H}^0}{\partial\lambda}}{\int \mathrm{d}\Gamma\, e^{-\beta\left(\mathcal{H}^0+\mathcal{H}^{sl}\right)}e^{\beta\mathcal{H}^{sl}}} - \left\langle\frac{\partial\mathcal{H}^0}{\partial\lambda}\right\rangle_{\lambda,a}\,. \end{aligned} \tag{S23}$$

Substituting in the first-order expansion $e^{\beta\mathcal{H}^{sl}} \approx 1 + \beta\mathcal{H}^{sl}$ gives, to leading order,

$$\begin{aligned}\frac{\partial W^0(\lambda)}{\partial\lambda} &\approx \frac{\int \mathrm{d}\Gamma\, e^{-\beta\left(\mathcal{H}^0+\mathcal{H}^{sl}\right)}\left(1+\beta\mathcal{H}^{sl}\right)\frac{\partial\mathcal{H}^0}{\partial\lambda}}{\int \mathrm{d}\Gamma\, e^{-\beta\left(\mathcal{H}^0+\mathcal{H}^{sl}\right)}\left(1+\beta\mathcal{H}^{sl}\right)} - \left\langle\frac{\partial\mathcal{H}^0}{\partial\lambda}\right\rangle_{\lambda,a} \\ &= \frac{\left\langle\left(1+\beta\mathcal{H}^{sl}\right)\frac{\partial\mathcal{H}^0}{\partial\lambda}\right\rangle_{\lambda,a}}{\left\langle 1+\beta\mathcal{H}^{sl}\right\rangle_{\lambda,a}} - \left\langle\frac{\partial\mathcal{H}^0}{\partial\lambda}\right\rangle_{\lambda,a} \\ &\approx \beta\left(\left\langle\mathcal{H}^{sl}\frac{\partial\mathcal{H}^0}{\partial\lambda}\right\rangle_{\lambda,a} - \left\langle\mathcal{H}^{sl}\right\rangle_{\lambda,a}\left\langle\frac{\partial\mathcal{H}^0}{\partial\lambda}\right\rangle_{\lambda,a}\right)\,. \end{aligned} \tag{S24}$$

This measures correlations between $\mathcal{H}^{sl}$ and $\partial\mathcal{H}^0/\partial\lambda$, which we argue are small. Under this assumption, ΔW is again given (albeit approximately) by Eq. (S21).

Approximating the surface as being of constant density in x and y allows the surface-liquid interaction energy to be written in terms of the per-liquid-molecule solid-liquid interaction energy $U^{sl}(z,\lambda)$ and phase-space-dependent liquid number density profile $n^l_\Gamma(z,\lambda)$,

$$\mathcal{H}^{sl}(\Gamma,\lambda) = A\int_0^\infty \mathrm{d}z\, U^{sl}(z,\lambda)n^l_\Gamma(z,\lambda)\,, \tag{S25}$$

where A is the interfacial area. This yields an expression in terms of the areal work of adhesion, w, as defined in the Young-Dupré equation (Eq. (3), main text),

$$\Delta w \equiv \frac{\Delta W}{A} = -\int_{\lambda_1}^{\lambda_2}\mathrm{d}\lambda\int_0^\infty \mathrm{d}z\,\left\langle\frac{\partial}{\partial\lambda}U^{sl}(z,\lambda)n^l_\Gamma(z,\lambda)\right\rangle_\lambda\,. \tag{S26}$$

Next, it is assumed that the solid-liquid interaction energy $U^{sl}(z,\lambda)$ is much more sensitively dependent on λ than is the density $n^l_\Gamma(z,\lambda)$. Then the product rule gives

$$\Delta w \approx -\int_{\lambda_1}^{\lambda_2}\mathrm{d}\lambda\int_0^\infty \mathrm{d}z\, n^l(z,\lambda)\frac{\partial U^{sl}(z,\lambda)}{\partial\lambda}\,, \tag{S27}$$

where $n^l(z,\lambda) = \left\langle n^l_\Gamma(z,\lambda)\right\rangle_\lambda$. The expectation value does not apply to the derivative $\partial U^{sl}(z,\lambda)/\partial\lambda$ because $U^{sl}(z,\lambda)$ is independent of the microstate Γ. For the case where

λ is the solid-liquid dispersion energy ϵ^{sl} (see Eq. (S8)),

$$\Delta w \approx -\int_{\epsilon_1^{sl}}^{\epsilon_2^{sl}} \mathrm{d}\epsilon^{sl} \int_0^\infty \mathrm{d}z\, n^l(z, \epsilon^{sl}) \frac{\partial U^{sl}(z, \epsilon^{sl})}{\partial \epsilon^{sl}} \,. \tag{S28}$$

The trapezoidal rule is applied to the integration over ϵ^{sl} at the integration boundaries alone, and the linearity of U^{sl} in ϵ^{sl} is used, giving

$$\frac{\Delta w}{\epsilon_2^{sl} - \epsilon_1^{sl}} \approx -\int_0^\infty \mathrm{d}z\, \frac{\partial U^{sl}(z, \epsilon^{sl})}{\partial \epsilon^{sl}} \frac{n_2^l(z) + n_1^l(z)}{2} \,, \tag{S29}$$

which can be calculated numerically from simulation data. Eq. (S29) can be used to check the consistency of the (purely geometrically) determined contact angle with the system energetics. From the Young-Dupré equation,

$$\frac{\Delta w}{\epsilon_2^{sl} - \epsilon_1^{sl}} = \gamma_{lv} \frac{\Delta \cos\theta_\infty}{\epsilon_2^{sl} - \epsilon_1^{sl}} \,. \tag{S30}$$

Fig. S6 (a–d) plots $\frac{\Delta \cos\theta_\infty}{\epsilon_2^{sl} - \epsilon_1^{sl}}$ from droplet simulations over $\frac{\Delta w}{\epsilon_2^{sl} - \epsilon_1^{sl}}$ calculated via Eq. (S29) for all systems considered. The black solid line is the linear function Eq. (S30) with the surface tensions γ_{lv} obtained from a pure planar water slab simulation, as given in Table S6. From Fig. S6 (a–d), it is clear that the change in contact angle is consistent with the change in free energy for all systems.

A similar analysis can be made when the number of layers is changed. Starting from Eq. (S27) and approximating $n^l(z, \lambda) \approx n^l(z)$ as being independent of λ allows the integral over λ to be taken, giving

$$\Delta w \approx -\int_0^\infty \mathrm{d}z\, \Delta U^{sl}(z) n^l(z) \approx -\int_0^\infty \mathrm{d}z\, \Delta U^{sl}(z) \frac{n^l(z, \lambda_2) + n^l(z, \lambda_1)}{2} \,, \tag{S31}$$

where $\Delta U^{sl}(z)$ is the difference in $U^{sl}(z, \lambda)$ made by adding or removing one or several graphene sheets. For example, going from one to two graphene sheets we have

$$\Delta U^{sl}(z) = U_G(z, -0.34 \text{ nm}) \,, \tag{S32}$$

where U_G is defined in Eq. (S10). The corresponding linear relationship based on the Young-Dupré equation is given by

$$\Delta w = \gamma_{lv} \Delta \cos\theta_\infty \,. \tag{S33}$$

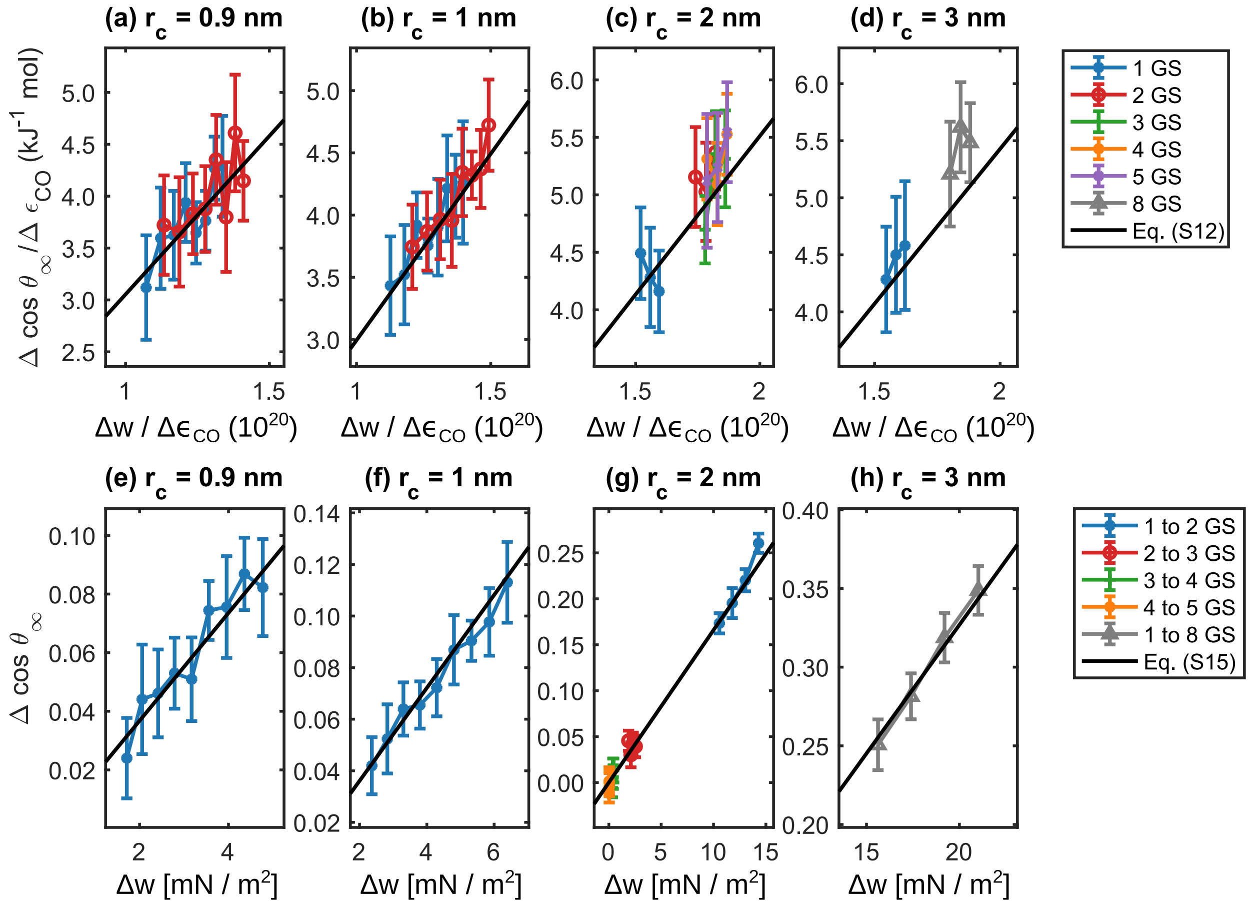

Figure S6: The change in contact angle related to the change in free energy. The Young-Dupré equation (black line) predicts a linear relationship where the proportionality is given by the liquid-vapor interfacial tension γ_{lv}. Top row: change due to a change of interaction strength, i.e., changing ϵ_{CO} according to Eq. (S30). Bottom row: change due to the change in the number of graphene layers according to Eq. (S33). Left to right: Different cutoff lengths r_c for the LJ interaction. (a) and (e) use a potential shift, all others use a force shift between $r_c - 0.1$ nm and r_c.

The final results for all systems are shown in Fig. S6 (e–h). In the case of short cutoffs, Fig. S6 (e) and (f), the interaction extends over at most two layers of graphene. Hence, only the difference between one and two layers yields non-zero contact angle differences and free energy differences. For Fig. S6 (g), the cutoff is 2 nm, resulting in differences in $\Delta \cos \theta_\infty$ up to 5 layers. In the last case, Fig. S6 (h), only systems with one layer or eight layers of graphene were simulated. Therefore only this difference could be calculated. For all systems, the agreement between measured differences and Eq. (S33) is very good, even for larger changes to the system, e.g. in Fig. S6 (h).

Finally, for the case where λ is the Lennard-Jones cutoff distance r_c (see Eq. (S9) and (S10)), approximating $n^l(z, r_c) \approx n^l(z)$ as being independent of r_c allows the integral over r_c in Eq. (S27) to be carried out explicitly, leaving

$$\Delta w \approx -\int_0^\infty \left[U^{sl}(z, r_2) - U^{sl}(z, r_1)\right] n^l(z) \mathrm{d}z \,. \tag{S34}$$

From the Young-Dupré Equation (Eq. (3) in the main text) we obtain a different expression for Δw, now in terms of the contact angle θ_∞ and liquid-vapor interfacial tension γ_{lv},

$$\Delta w = \gamma_{lv}(r_2)(1 + \cos\theta_\infty(r_2)) - \gamma_{lv}(r_1)(1 + \cos\theta_\infty(r_1)) \,. \tag{S35}$$

Finally, Eqs. (S34) and (S35) can be combined to give

$$\cos\theta_\infty(r_2) \approx \frac{\gamma_{lv}(r_1)(1 + \cos\theta_\infty(r_1)) - \int_0^\infty \left[U^{sl}(z, r_2) - U^{sl}(z, r_1)\right] n^l(z) \mathrm{d}z}{\gamma_{lv}(r_2)} - 1 \,, \tag{S36}$$

which is reproduced in the main text as Eq. (4).

S9 Graphene Force Fields for Several Water Models

Graphene-/graphite-water force fields are also developed for several popular non-polarizable, rigid water models in addition to SPC/E.[28] Included are 3-point models TIP3P[36] and OPC3,[37] 4-point models TIP4P-Ew,[38] TIP4P/2005,[39] and OPC,[40] and the 5-point model TIP5P-E.[41] As is done throughout this work, σ_{CO} is set to 0.3367 nm. The LJ forces are smoothly switched off between 1.0 and 1.2 nm. Planar simulations of water in vacuum (which results in a liquid-vapor system), and water on a single graphene sheet (which results in a solid-liquid-vapor system) with $\epsilon_{\mathrm{CO}} \in \{0.35, 0.4, 0.45, 0.5, 0.55\}$ kJ/mol, are carried out. In all simulations, 1024 water molecules are simulated in a box of size $L_x \approx 3.197$ nm, $L_y = 2.982$ nm, and $L_z = 25$ nm. Where graphene is included, it consists of a single graphene sheet of 364 spatially frozen carbon atoms. The production run duration of all simulations is 100 ns. Simulation details not specified here are consistent with those given in Section S2.

The liquid-vapor surface tension γ_{lv} of each water model is calculated from the pressure tensor of the liquid-vapor simulation, and the resulting values are presented in Table S7. The vacuum wetting coefficient,

$$k_{\mathrm{vac}} \equiv \frac{\gamma_{s\,\mathrm{vac}} - \gamma_{sl}}{\gamma_{lv}}\,, \tag{S37}$$

is extracted via Eq. (S52), which follows from Eq. (11) in the main text. We assume that for the relevant regime (60°–80°), the vacuum wetting coefficient is a good approximation of the wetting coefficient $k = \cos\theta_\infty$, i.e., that $\gamma_{s\,\mathrm{vac}} \approx \gamma_{sv}$. This assumption breaks down once surface-liquid interactions are strong enough for an equilibrium vapor phase to spontaneously build a significant film on the surface.[42] We are confident the assumption is a good one because of the excellent agreement between contact angles calculated via the pressure-tensor and droplet methods, even for very hydrophilic surfaces, which can be seen in Figure 4 (d) in the main text. The extracted vacuum wetting coefficients are plotted over ϵ_{CO} in Fig. S7. Quadratic fits of Eq. (S12),

$$k_{\mathrm{vac}}(\epsilon_{\mathrm{CO}}) = a\epsilon_{\mathrm{CO}}^2 + b\epsilon_{\mathrm{CO}} + c\,,$$

are carried out and are also shown in Fig. S7. The resulting fit parameters are given in Table S7. Finally, the ϵ_{CO} values where the fits give cos(80°) and cos(60°) are calculated. These are marked as + and × symbols, respectively, in Fig. S7, and are given in Table S7.

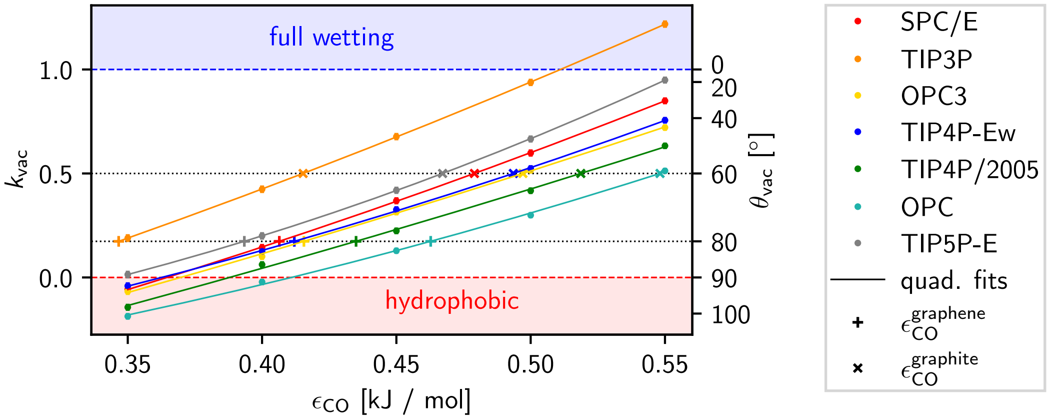

Figure S7: Vacuum wetting coefficients k_{vac} as a function of ϵ_{CO} for several popular water models (dots), where $\sigma_{CO} = 0.3367$ nm and LJ forces are smoothly switched off between 1.0 and 1.2 nm. Also shown are quadratic fits of the data (lines). The k_{vac} values that correspond to the graphene and graphite contact angles of 80° and 60° are shown as horizontal dotted lines, and their intersections with the fitted curves give the ϵ_{CO} values needed to simulate graphene and graphite; these are marked with + and × symbols, respectively. The results are presented in Table S7.

Table S7: Liquid-vapor surface tensions, quadratic fit parameters, and carbon-oxygen dispersion energies that reproduce graphene- and graphite-water contact angles, for several water models

water model	γ_{lv} [mN/m]	a [mol^2/kJ^2]	b [mol/kJ]	c	$\epsilon_{CO}^{graphene}$ [kJ/mol]	$\epsilon_{CO}^{graphite}$ [kJ/mol]
SPC/E[28]	55.90 ± 0.44	2.95989	1.87273	-1.07639	0.406423	0.479049
TIP3P[36]	47.29 ± 0.41	2.81949	2.60496	-1.06800	0.346613	0.415274
OPC3[37]	59.97 ± 0.45	1.67544	2.47341	-1.14361	0.415578	0.497114
TIP4P-Ew[38]	58.56 ± 0.45	3.67745	0.67298	-0.72786	0.412005	0.493531
TIP4P/2005[39]	63.02 ± 0.47	1.59678	2.37743	-1.16265	0.434991	0.518667
OPC[40]	69.09 ± 0.47	3.44581	0.33896	-0.72119	0.462782	0.548159
TIP5P-E[41]	51.83 ± 0.43	6.35144	-1.04405	-0.39859	0.393400	0.467202

S10 Contact Angles from Pressure Tensors

The fundamental thermodynamic relation for deformed bodies gives the total differential for the internal energy $\mathcal{U}$ (using the Einstein summation convention),

$$\begin{aligned} \mathrm{d}\mathcal{U} &= T\mathrm{d}S - V_0 P_{ij}\mathrm{d}\varepsilon_{ij} \\ &= T\mathrm{d}S + \mathrm{d}\mathcal{W}\,, \end{aligned} \tag{S38}$$

where T is the temperature, S the entropy, P_{ij} the pressure tensor, V_0 the volume of the undeformed system, ε_{ij} the strain tensor, and $\mathcal{W}$ the mechanical work done by the deformation against internal stresses. The differential for the free energy $\mathcal{F}$ is similarly given by

$$\mathrm{d}\mathcal{F} = \mathrm{d}\mathcal{U} - \mathrm{d}(ST) = -S\mathrm{d}T + \mathrm{d}\mathcal{W}\,. \tag{S39}$$

The strain tensor is unitless, given to first order by

$$\varepsilon_{ij} = \frac{1}{2}\left(\frac{\partial(x_i' - x_i)}{\partial x_j} + \frac{\partial(x_j' - x_j)}{\partial x_i}\right)\,,$$

where a particle at x_i is moved to x_i' by the deformation. Letting the Cartesian coordinates correspond to the principle axes of the strain gives a diagonal ε_{ij} with $\varepsilon_{ij} = 0$ for $i \neq j$ and

$$\varepsilon_{ii} = \frac{\partial(x_i' - x_i)}{\partial x_i}\,.$$

Since we are interested in the interfacial tension in the xy-plane, we additionally restrict the deformation in the xy-plane to be isotropic, i.e., $\varepsilon_{xx} = \varepsilon_{yy} \equiv \varepsilon_T$, where ε_T is the strain tangential to the xy-plane, which gives

$$\mathrm{d}\mathcal{W} = -V_0\left(P_{xx} + P_{yy}\right)\mathrm{d}\varepsilon_T - V_0 P_{zz}\mathrm{d}\varepsilon_{zz}\,. \tag{S40}$$

We consider only systems consisting of bulk phases separated by interfaces in the xy-plane and no triphasic lines, so $\mathrm{d}\mathcal{W}$ can be written as

$$\mathrm{d}\mathcal{W} = -p\mathrm{d}V + \gamma\mathrm{d}A\,, \tag{S41}$$

where p is the pressure in the bulk, γ the interfacial tension of the system in the xy-plane, and V and A the deformation-dependent system volume and area in the xy-plane. This equation amounts to a definition of γ, and the explicit V dependence is important here because γ is an interfacial tension and should therefore exclude energetic contributions from

volumetric changes. Let L_x, L_y and L_z be the dimensions of the undeformed system, which are transformed to L'_x, L'_y, and L'_z by the deformation. The area and volume can be written

$$\begin{aligned} A &= L'_x L'_y = \left(1+\varepsilon_T\right)^2 L_x L_y \approx \left(1+2\varepsilon_T\right) L_x L_y \\ V &= L'_x L'_y L'_z = \left(1+\varepsilon_T\right)^2 \left(1+\varepsilon_{zz}\right) L_x L_y L_z \approx \left(1+2\varepsilon_T+\varepsilon_{zz}\right) V_0 \,, \end{aligned}$$

where in the last step on each line, the expression is approximated to first order in ε_T and ε_{zz}. The total differentials can be directly calculated as

$$\mathrm{d}A = 2L_x L_y \mathrm{d}\varepsilon_T \qquad \text{and} \qquad \mathrm{d}V = V_0\left(2\mathrm{d}\varepsilon_T + \mathrm{d}\varepsilon_{zz}\right) \,,$$

which, when substituted into Eq. (S40) give

$$\mathrm{d}\mathcal{W} = -P_{zz}\mathrm{d}V + L_z\left(P_{zz} - \frac{P_{xx}+P_{yy}}{2}\right)\mathrm{d}A \,. \tag{S42}$$

Comparison of Eqs. (S38), (S39), (S41), and (S42) reveals

$$\gamma = \left(\frac{\partial\mathcal{U}}{\partial A}\right)_{S,V} = \left(\frac{\partial\mathcal{F}}{\partial A}\right)_{T,V} = \left(\frac{\partial\mathcal{W}}{\partial A}\right)_{V} = L_z\left(P_{zz} - \frac{P_{xx}+P_{yy}}{2}\right) \,, \tag{S43}$$

the last of which is reproduced in the main text as Eq. (7).

Decomposition of System Interfacial Tensions for Contact Angle Calculation: The total system interfacial tension can be decomposed into kinetic and virial (arising from the forces and positions) contributions for each particle type. The virial contributions can be further decomposed according to which pairwise interaction is involved, e.g., the interfacial tension for a solid-liquid system is given by

$$\gamma = \gamma_S^{sl} + \gamma_S^{ss} + \gamma_S^{\mathrm{kin}} + \gamma_L^{sl} + \gamma_L^{ll} + \gamma_L^{\mathrm{kin}} \,, \tag{S44}$$

where the subscripts S and L indicate that contributions arise from deformations and/or kinetic energy of solid and liquid atoms, respectively, and the superscripts indicate for the virial terms the interactions involved, denoted by the two phases between which they act (e.g., *sl* indicates solid-liquid interactions), or "kin" for kinetic terms. Here, γ_L^{sl}, γ_L^{ll}, and γ_L^{kin} encompass contributions due to deformations and kinetic energy of all atoms of the liquid *type* (e.g., all water molecules in the system), which also includes vapor. The notations γ_{sl}, γ_{sv} and γ_{lv} from the main text are reserved for tensions of single solid-liquid, solid-vapor, and liquid-vapor interfaces, respectively. Consider the systems in Fig. 4 in the main text.

For system a, appearing in Fig. 4 (a), the liquid-atom contributions must exactly equal the solid-liquid and liquid-vapor interfacial tensions. Together with Eq. (S44), this gives

$$\gamma_L^{sl} + \gamma_L^{ll} + \gamma_L^{\mathrm{kin}} = \gamma_{sl} + \gamma_{lv} \tag{S45}$$

$$\Rightarrow \quad \gamma_a = \gamma_{lv} + \gamma_{sl} + \gamma_S^{sl} + \gamma_S^{ss} + \gamma_S^{\mathrm{kin}}\,, \tag{S46}$$

which is reproduced in the main text as Eq. (8). For system b (Fig. 4 (b), main text), there are two liquid-vapor interfaces, which, without any decomposition, gives

$$\gamma_b = 2\gamma_{lv}\,, \tag{S47}$$

which is reproduced in the main text as Eq. (9). Eq. (S44) also applies to the last system, system c (Fig. 4 (c), main text), but since the terms are generally not equal to those for system a, we denote them with an additional subscript (v). Here, the contributions from the atoms in the vapor must exactly equal the solid-vapor interfacial tension, i.e.,

$$\gamma_{L\,(v)}^{sl} + \gamma_{L\,(v)}^{ll} + \gamma_{L\,(v)}^{\mathrm{kin}} = \gamma_{sv}$$

$$\Rightarrow \quad \gamma_c = \gamma_{sv} + \gamma_{S\,(v)}^{sl} + \gamma_{S\,(v)}^{ss} + \gamma_{S\,(v)}^{\mathrm{kin}}\,. \tag{S48}$$

It is expected that $\gamma_S^{sl} \gg \gamma_{S\,(v)}^{sl}$ because the liquid phase is much denser than the vapor phase, and that generally, $\gamma_S^{ss} \neq \gamma_{S\,(v)}^{ss}$ because a solid configuration may be slightly modified by the presence of the liquid itself, so the deformation of the solid will have a slightly different influence on the solid-solid interaction energy. Substituting Eq. (S46), (S47), and (S48) into Young's equation (Eq. (6), main text) yields

$$\cos\theta_\infty = \frac{\gamma_{sv} - \gamma_{sl}}{\gamma_{lv}}$$

$$\Rightarrow \quad \cos\theta_\infty = 2\frac{\gamma_c - \gamma_{S\,(v)}^{ss} - \gamma_{S\,(v)}^{sl} - \gamma_{S\,(v)}^{\mathrm{kin}} - \gamma_a + \gamma_S^{sl} + \gamma_S^{ss} + \gamma_S^{\mathrm{kin}}}{\gamma_b} + 1\,. \tag{S49}$$

For a liquid with a low vapor pressure such as water, the contribution of the vapor might be taken to be negligible, in which case $\gamma_{S\,(v)}^{sl} = 0$. For a stiff solid whose configuration is unaffected by the presence of the liquid, $\gamma_{S\,(v)}^{ss} = \gamma_S^{ss}$, and $\gamma_{S\,(v)}^{\mathrm{kin}} = \gamma_S^{\mathrm{kin}}$. For most systems it should hold that $\gamma_{S\,(v)}^{\mathrm{kin}} = \gamma_S^{\mathrm{kin}}$ due to equipartition, and for a surface consisting of spatially frozen atoms, $\gamma_{S\,(v)}^{\mathrm{kin}} = \gamma_S^{\mathrm{kin}} = 0$. Under these assumptions, the tension of system c can be written

$$\gamma_c = \gamma_{sv} + \gamma_S^{ss} + \gamma_S^{\mathrm{kin}}\,, \tag{S50}$$

which is reproduced in the main text as Eq. (10). Under the low vapor pressure assumption, it might also be expected that $\gamma_{sv} = 0$, but we formally leave it in. Substituting Eq. (S50) instead of Eq. (S48) into Young's equation gives

$$\cos\theta_\infty = 2\frac{\gamma_c - \gamma_a + \gamma_S^{sl}}{\gamma_b} + 1\,, \tag{S51}$$

which is reproduced in the main text as Eq. (11). In our case, $\gamma_c = 0$, because there are no interactions among carbons and there would be no waters present in the simulation due to the low vapor pressure of water, thus,

$$\cos\theta_\infty = 2\frac{-\gamma_a + \gamma_S^{sl}}{\gamma_b} + 1\,. \tag{S52}$$

The system interfacial tensions γ_a, γ_b, and γ_c can be obtained directly from the pressure tensors of the three planar simulations, so to calculate contact angles, γ_S^{sl} is required.

Direct Calculation of $\boldsymbol{\gamma_S^{sl}}$ from Densities and Interaction Potentials: There are multiple choices for volume-preserving deformations that are isotropic in the xy-plane. We choose the deformation with the strain tensor

$$\varepsilon = \begin{pmatrix} \lambda/2 & 0 & 0 \\ 0 & \lambda/2 & 0 \\ 0 & 0 & -\lambda \end{pmatrix}\,,$$

for which the differential of A becomes $\mathrm{d}A = L_x L_y \mathrm{d}\lambda$. This deformation is volume preserving to first order, i.e., for infinitesimal deformations. Together with Eq. (S40), this yields

$$\begin{aligned} \mathrm{d}\mathcal{W} &= -V_0\left(P_{xx} + P_{yy}\right)\frac{\mathrm{d}\lambda}{2} + V_0 P_{zz}\mathrm{d}\lambda \\ &= L_z\left(P_{zz} - \frac{P_{xx} + P_{yy}}{2}\right)\mathrm{d}A\,, \end{aligned}$$

consistent with Eq. (S42) with $\mathrm{d}V = 0$. The corresponding deformation is given by

$$\Phi(\boldsymbol{r}, \lambda) = \begin{pmatrix} 1 + \lambda/2 & 0 & 0 \\ 0 & 1 + \lambda/2 & 0 \\ 0 & 0 & 1 - \lambda \end{pmatrix} \cdot \boldsymbol{r}\,.$$

Some useful derivatives are

$$
\begin{aligned}
\partial_\lambda \Phi(\boldsymbol{r},\lambda)|_{\lambda=0} &= \underbrace{\begin{pmatrix} 1/2 & 0 & 0 \\ 0 & 1/2 & 0 \\ 0 & 0 & -1 \end{pmatrix}}_{=M} \cdot \boldsymbol{r} = \mathrm{M}\cdot\boldsymbol{r}\,, \\
\partial_i \Phi_j(\boldsymbol{r},\lambda)|_{\lambda=0} &= \left.\begin{pmatrix} 1+\lambda/2 & 0 & 0 \\ 0 & 1+\lambda/2 & 0 \\ 0 & 0 & 1-\lambda \end{pmatrix}\right|_{\lambda=0} = \mathbb{I}_3\,, \\
\det \partial_i \Phi_j(\boldsymbol{r},\lambda)|_{\lambda=0} &= 1\,.
\end{aligned}
$$

Consider now a system consisting of a solid with a flat surface and a liquid adsorbed on the surface, i.e., system a in Fig. 4 (a) in the main text. Starting from Eq. (S43), the total interfacial tension of the system in the xy-plane is given by

$$
\begin{aligned}
\gamma &= \left.\left(\frac{\partial \mathcal{U}}{\partial A}\right)_{S,V}\right|_{A=L_xL_y} \\
&= (\partial_A \lambda)\; \partial_\lambda \mathcal{U}|_{\lambda=0} \\
&= \frac{1}{L_xL_y}\,\partial_\lambda \mathcal{U}|_{\lambda=0} \\
&= \frac{1}{L_xL_y}\,\partial_\lambda \mathcal{U}_0|_{\lambda=0} + \underbrace{\frac{1}{L_xL_y}\,\partial_\lambda \mathcal{U}^{sl}|_{\lambda=0}}_{\gamma_S^{sl}+\gamma_L^{sl}}\,,
\end{aligned}
\tag{S53}
$$

where $\mathcal{U} = \mathcal{U}_0 + \mathcal{U}^{sl}$, with $\mathcal{U}^{sl}$ the energy of all solid-liquid interactions, and the sum $\gamma_S^{sl} + \gamma_L^{sl}$ constitutes the contribution to the surface tension from these interactions (see Eq. (S44)). The deformation Φ is assumed to be adiabatic and isochoric, so ∂_λ in the second line is consistent with the subscripts S and V in the first line. Let the solid-liquid interactions consist only of interactions between one species of atom of the solid, and one of the liquid. Let these interactions be mediated by the interaction potential $U(\boldsymbol{r}^l, \boldsymbol{r}^s)$ where $\boldsymbol{r}^l$ and $\boldsymbol{r}^s$ are positions within the liquid and solid phases respectively. The solid-liquid interaction energy per simulation box (with periodic boundary conditions taken into account) is given by

$$
\mathcal{U}^{sl} = \int_{\mathbb{R}^3} \mathrm{d}\boldsymbol{r}^s \int_{\vec{L}} \mathrm{d}\boldsymbol{r}^l\, n(\boldsymbol{r}^l, \boldsymbol{r}^s) U(\boldsymbol{r}^l, \boldsymbol{r}^s)\,,
$$

where $\vec{L} = (L_x, L_y, L_z)$ indicates the unit cell dimensions, $\int_{\vec{L}} \mathrm{d}\boldsymbol{r}$ an integral over a single unit cell, and $n(\boldsymbol{r}^l, \boldsymbol{r}^s)$ the pairwise solid-liquid atomic number density. Due to particle

conservation, the pairwise number density upon deformation n_d is given by

$$n_d(\boldsymbol{r}^l, \boldsymbol{r}^s, \lambda) = \frac{n(\Phi^{-1}(\boldsymbol{r}^l, \lambda), \Phi^{-1}(\boldsymbol{r}^s, \lambda))}{\det \partial_j \Phi_i(\boldsymbol{r}^l, \lambda) \det \partial_j \Phi_i(\boldsymbol{r}^s, \lambda)} = n(\Phi^{-1}(\boldsymbol{r}^l, \lambda), \Phi^{-1}(\boldsymbol{r}^s, \lambda))\,.$$

Using this result, the effect of the deformation on $\mathcal{U}^{sl}$ can be recast as a transformation of the potential U only,

$$\begin{aligned}
\mathcal{U}^{sl}(\lambda) &= \int_{\mathbb{R}^3} \mathrm{d}\boldsymbol{r}^s \int_{\Phi(\vec{L},\lambda)} \mathrm{d}\boldsymbol{r}^l\, n_d(\boldsymbol{r}^l, \boldsymbol{r}^s, \lambda) U(\boldsymbol{r}^l, \boldsymbol{r}^s) \\
&= \int_{\mathbb{R}^3} \mathrm{d}\boldsymbol{r}^s \int_{\Phi(\vec{L},\lambda)} \mathrm{d}\boldsymbol{r}^l\, n(\Phi^{-1}(\boldsymbol{r}^l, \lambda), \Phi^{-1}(\boldsymbol{r}^s, \lambda)) U(\boldsymbol{r}^l, \boldsymbol{r}^s) \\
&= \int_{\mathbb{R}^3} \mathrm{d}\boldsymbol{r}^s \int_{\vec{L}} \mathrm{d}\boldsymbol{r}^l\, n(\boldsymbol{r}^l, \boldsymbol{r}^s) U(\Phi(\boldsymbol{r}^l, \lambda), \Phi(\boldsymbol{r}^s, \lambda))\,.
\end{aligned}$$

Next we compute the derivative

$$\partial_\lambda \mathcal{U}^{sl}(\lambda)\big|_{\lambda=0} = \int_{\mathbb{R}^3} \mathrm{d}\boldsymbol{r}^s \int_{\vec{L}} \mathrm{d}\boldsymbol{r}^l\, n(\boldsymbol{r}^l, \boldsymbol{r}^s) \left[\underbrace{(\partial_i^l U(\boldsymbol{r}^l, \boldsymbol{r}^s)) \cdot M_{ij} r_j^l}_{\text{deformation of liquid}} + \underbrace{(\partial_i^s U(\boldsymbol{r}^l, \boldsymbol{r}^s)) \cdot M_{ij} r_j^s}_{\text{deformation of solid}} \right]. \tag{S54}$$

Combining Eqs. (S53) and (S54) gives

$$\begin{aligned}
\gamma_L^{sl} &= \frac{1}{L_x L_y} \int_{\mathbb{R}^3} \mathrm{d}\boldsymbol{r}^s \int_{\vec{L}} \mathrm{d}\boldsymbol{r}^l\, n(\boldsymbol{r}^l, \boldsymbol{r}^s) (\partial_i^l U(\boldsymbol{r}^l, \boldsymbol{r}^s)) \cdot M_{ij} r_j^l \quad \text{and} \\
\gamma_S^{sl} &= \frac{1}{L_x L_y} \int_{\mathbb{R}^3} \mathrm{d}\boldsymbol{r}^s \int_{\vec{L}} \mathrm{d}\boldsymbol{r}^l\, n(\boldsymbol{r}^l, \boldsymbol{r}^s) (\partial_i^s U(\boldsymbol{r}^l, \boldsymbol{r}^s)) \cdot M_{ij} r_j^s\,.
\end{aligned}$$

We are only interested in finding an explicit expression for γ_S^{sl}. We assume the solid-liquid pairwise particle density to be factorizable (for our system this holds because the atoms of the solid are spatially frozen) and that the two densities are well approximated as being constant along x and y, which together give

$$n(\boldsymbol{r}^l, \boldsymbol{r}^s) = n^l(z^l) n^s(z^s)\,.$$

This can be substituted into the previous result for γ_S^{sl}, yielding

$$\gamma_S^{sl} = \frac{1}{L_x L_y} \int_{\vec{L}} \mathrm{d}\boldsymbol{r}^l\, n^l(z^l) \int_{\mathbb{R}^3} \mathrm{d}\boldsymbol{r}^s\, n^s(z^s) (\partial_i^s U(\boldsymbol{r}^l, \boldsymbol{r}^s)) \cdot M_{ij} r_j^s\,.$$

Next, we solve just the integral over $\boldsymbol{r}^s$,

$$
\begin{aligned}
&\int_{\mathbb{R}^3} \mathrm{d}\boldsymbol{r}^s\, n^s(z^s)(\partial_i^s U(\boldsymbol{r}^l, \boldsymbol{r}^s)) \cdot M_{ij} r_j^s \\
&= \int_{\mathbb{R}^3} \mathrm{d}\boldsymbol{r}^s\, n^s(z^s) \left[\frac{x^s}{2}\partial_x^s U(\boldsymbol{r}^l, \boldsymbol{r}^s) + \frac{y^s}{2}\partial_y^s U(\boldsymbol{r}^l, \boldsymbol{r}^s) - z^s \partial_z^s U(\boldsymbol{r}^l, \boldsymbol{r}^s)\right] \\
&= \frac{1}{2}\int_{-\infty}^{\infty} \mathrm{d}z^s\, n^s(z^s) \int_{\mathbb{R}^2} \mathrm{d}x^s \mathrm{d}y^s\, \left[x^s \partial_x^s U(\boldsymbol{r}^l, \boldsymbol{r}^s) + y^s \partial_y^s U(\boldsymbol{r}^l, \boldsymbol{r}^s)\right] - \int_{\mathbb{R}^3} \mathrm{d}\boldsymbol{r}^s\, n^s(z^s) z^s \partial_z^s U(\boldsymbol{r}^l, \boldsymbol{r}^s) \\
&= \frac{1}{2}\int_{-\infty}^{\infty} \mathrm{d}z^s n^s(z^s) \underbrace{\int_{\mathbb{R}^2} \mathrm{d}x^s \mathrm{d}y^s\, \left[\partial_x^s (x^s U(\boldsymbol{r}^l, \boldsymbol{r}^s)) + \partial_y^s (y^s U(\boldsymbol{r}^l, \boldsymbol{r}^s))\right]}_{=0} \\
&\quad - \frac{1}{2}\int_{-\infty}^{\infty} \mathrm{d}z^s\, n^s(z^s) \int_{\mathbb{R}^2} \mathrm{d}x^s \mathrm{d}y^s\, \left[U(\boldsymbol{r}^l, \boldsymbol{r}^s) + U(\boldsymbol{r}^l, \boldsymbol{r}^s)\right] - \int_{\mathbb{R}^3} \mathrm{d}\boldsymbol{r}^s\, n^s(z^s) z^s \partial_z^s U(\boldsymbol{r}^l, \boldsymbol{r}^s) \\
&= -\int_{\mathbb{R}^3} \mathrm{d}\boldsymbol{r}^s\, n^s(z^s) \left[U(\boldsymbol{r}^l, \boldsymbol{r}^s) + z^s \partial_z^s U(\boldsymbol{r}^l, \boldsymbol{r}^s)\right] .
\end{aligned}
$$

The integral denoted as equal zero vanishes due to Gauss' theorem, and $U = 0$ for distances greater than the cutoff radius r_c.[1] This gives

$$
\gamma_S^{sl} = \frac{-1}{L_x L_y} \int_{\vec{L}} \mathrm{d}\boldsymbol{r}^l\, n^l(z^l) \int_{\mathbb{R}^3} \mathrm{d}\boldsymbol{r}^s\, n^s(z^s) \left[U(\boldsymbol{r}^l, \boldsymbol{r}^s) + z^s \partial_z^s U(\boldsymbol{r}^l, \boldsymbol{r}^s)\right] ,
$$

which is reproduced in the main text as Eq. (12). Taking the solid to be composed of N_G continuous graphene sheets positioned at z_i, each with an average areal density of n_A, i.e., $n^s(z^s) = n_A \sum_{i=1}^{N_G} \delta(z^s - z_i)$, we obtain for the inner integral over $\boldsymbol{r}^s$,

$$
\begin{aligned}
-\int_{\mathbb{R}^3} \mathrm{d}\boldsymbol{r}^s\, n^s(z^s) \left[U(\boldsymbol{r}^l, \boldsymbol{r}^s) + z^s \partial_z^s U(\boldsymbol{r}^l, \boldsymbol{r}^s)\right] &= -\sum_{i=1}^{N_G} \left[U_G(z^l, z_i) + z_i \partial_{z_i} U_G(z^l, z_i)\right] \\
&= -\sum_{i=1}^{N_G} \left[U_G(z^l, z_i) - z_i \partial_z^l U_G(z^l, z_i)\right] .
\end{aligned}
$$

Finally, substituting this into the previous expression for γ_S^{sl} yields

$$
\gamma_S^{sl} = \sum_{i=1}^{N_G} \int_0^{L_z} \mathrm{d}z^l\, n^l(z^l) \left[z_i \partial_z^l U_G(z^l, z_i) - U_G(z^l, z_i)\right] . \tag{S55}
$$

[1] This is also true if the interaction potential decays faster than $1/r$.

S11 Pressure Tensor vs. Virial

The pressure tensor consists of kinetic and virial contributions and is given by

$$P_{ij} = \frac{2}{V}\left(E_{ij} - \Theta_{ij}\right) , \tag{S56}$$

where $E_{ij} = \left\langle \frac{1}{2} \sum_\alpha m^\alpha v_i^\alpha v_j^\alpha \right\rangle$ is the kinetic energy tensor and Θ_{ij} is the virial. According to the equipartition theorem, the kinetic energy tensor should be a diagonal matrix with equal entries on the diagonal. Hence, the kinetic contribution should cancel out exactly when calculating the system interfacial tension using Eq. (7) in the main text and be given by

$$\gamma = -\frac{2L_z}{V}\left(\Theta_{zz} - \frac{\Theta_{xx} + \Theta_{yy}}{2}\right) . \tag{S57}$$

Fig. S8 shows the surface tension calculated from Eq. (7) in the main text and Eq. (S57) for a graphene-water system (a–c) and a pure water system (d–f) for both flexible and rigid water molecules. The two equations clearly agree only for flexible water. Fig. S8 (c) and (f) in the right column are included to rule out the possibility that the deviation could be due to the smaller timestep required for flexible water and the resulting improvement in numerical accuracy. The reason for the deviation is the difference in the diagonal elements of the kinetic energy tensor shown in Fig. S9, which shows that the diagonal elements are only identical in the case of flexible water.

To obtain some intuition, consider a rigid diatomic molecule, i.e., a diatomic molecule whose bond length is constrained, that is trapped by a harmonic potential such that the bond axis is almost exclusively aligned with the z-axis. The average translational kinetic energy is separable and is equal to $k_BT/2$ for each dimension. Due to the harmonic constraint, the two rotational modes are restricted to oscillations in the x direction (rotation around the y-axis) and the y direction (rotation around the x-axis). Hence the rotational degrees of freedom lead to a contribution of $k_BT/2$ only for E_{xx} and E_{yy} and the total kinetic energy tensor is given by $E_{xx} = E_{yy} = k_BT$ and $E_{zz} = k_BT/2$. This simple example shows that constraints in bond length can lead to kinetic energy tensors whose diagonal elements are not equal in each direction. The surface tensions from Eq. (S57) are therefore not the correct surface tensions because they do not include the kinetic contributions. This discrepancy was noted previously by Sega *et al.*[43]

Sedlmeier *et al.*[44] and Sendner *et al.*[45] use surface tensions obtained from Eq. (S57) to calculate the contact angle for water on diamond and obtain very good agreement with the results obtained from droplet simulations. We compare the contact angles obtained using the

pressure tensor (Eq. (7), main text) and the virial only (Eq. (S57)) to the droplet simulations in Fig. S10. Using only the virial tensor with (purple) and without (green) accounting for γ_S^{sl} leads to wrong results. Only the surface tension obtained from the total pressure tensor accounting for γ_S^{sl} are correct.

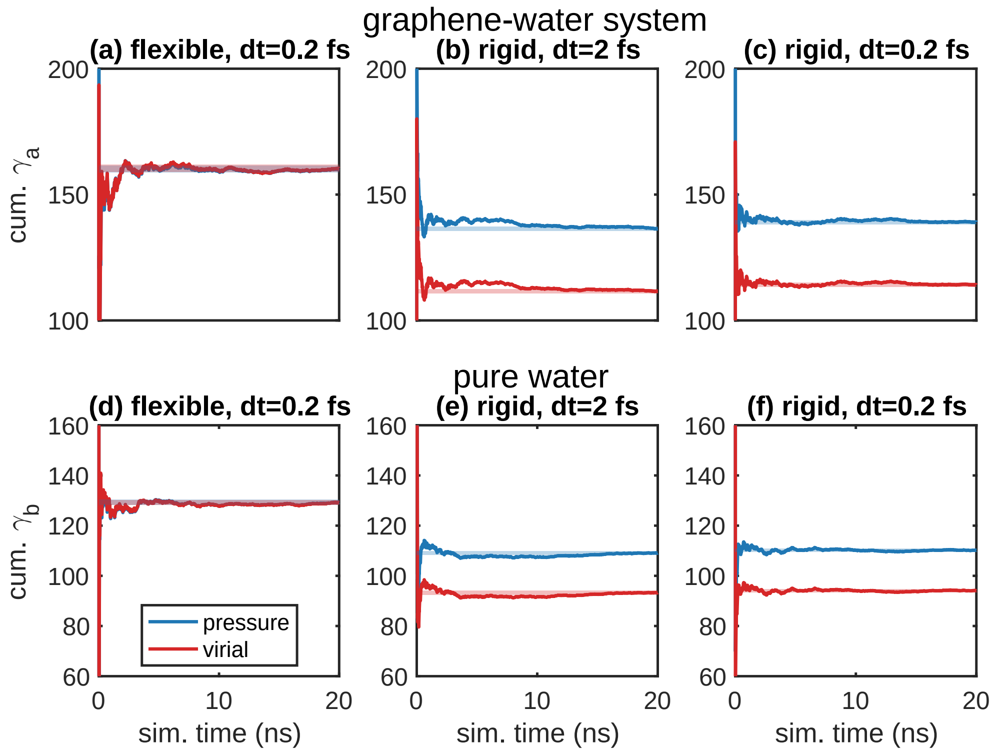

Figure S8: Cumulative average of the surface tension calculated using the full pressure tensor (Eq. (7) in the main text) and only the virial tensor (Eq. (S57)). Top: Planar graphene-water system. Bottom: Planar water slab. Left column: Flexible water and integration timestep 0.2 fs. Middle column: Rigid water and integration timestep 2 fs. Right column: Rigid water and integration timestep 0.2 fs

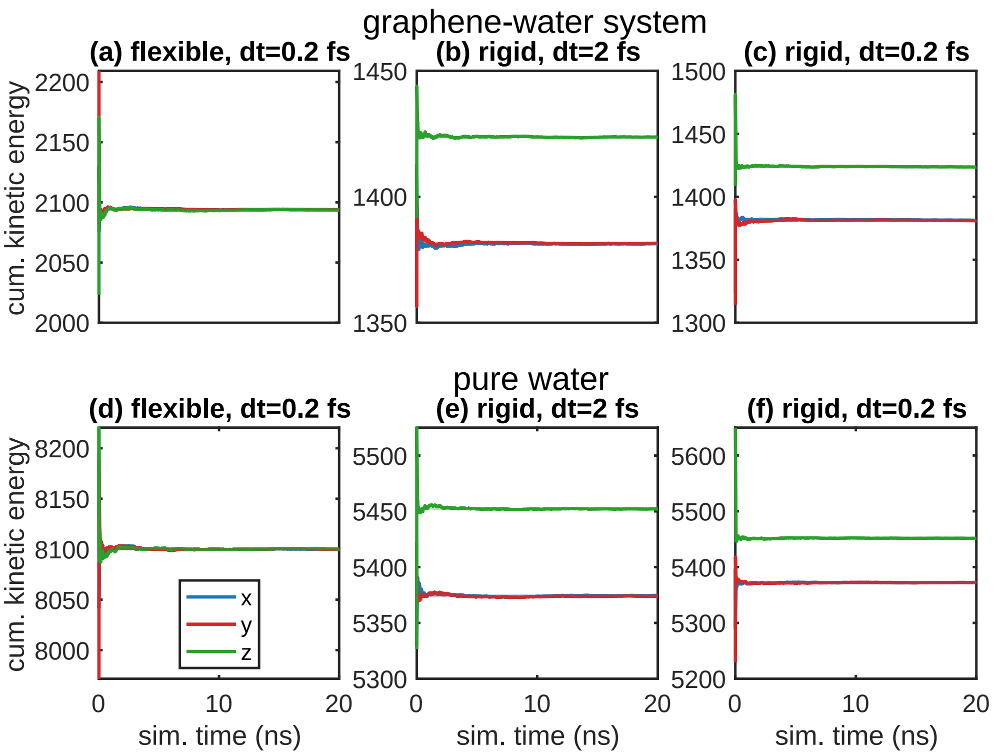

Figure S9: Cumulative average of the diagonal kinetic energy tensor elements. Top: Planar graphene-water system. Bottom: Planar water slab. Left column: Flexible water and integration timestep 0.2 fs. Middle column: Rigid water and integration timestep 2 fs. Right column: Rigid water and integration timestep 0.2 fs

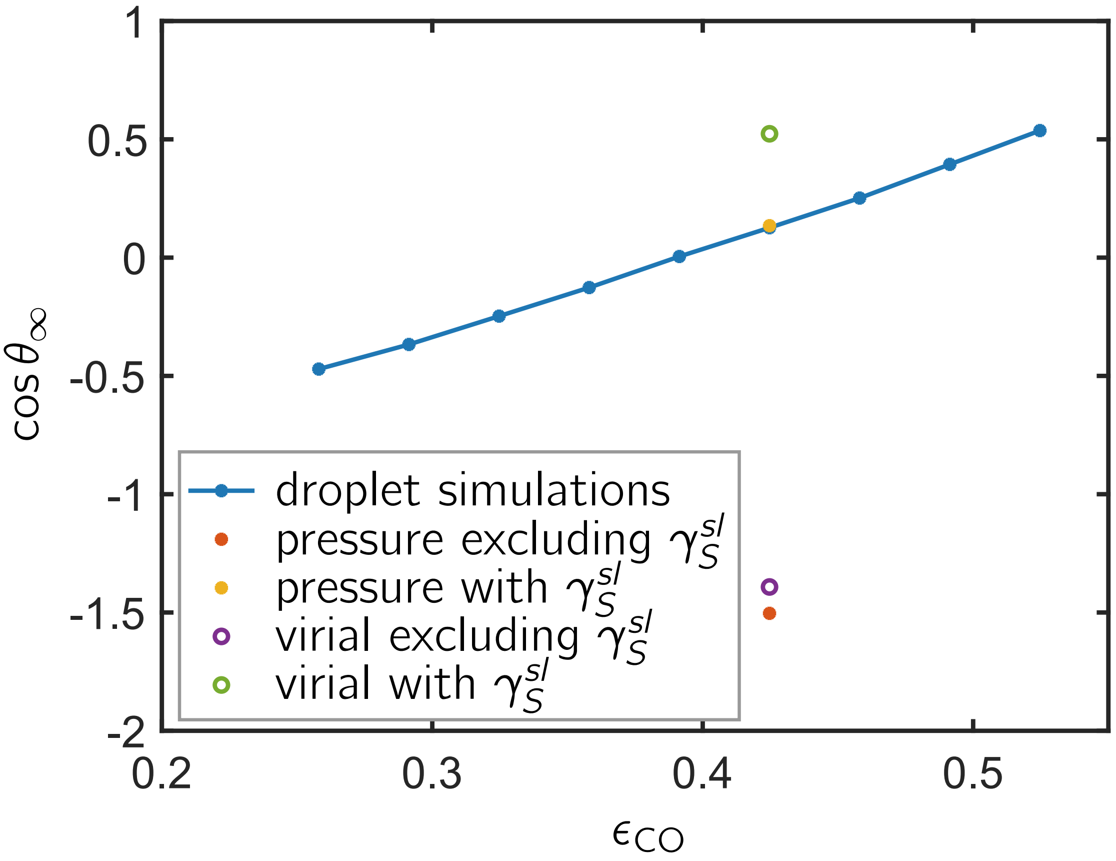

Figure S10: The cosine of the macroscopic contact angle θ_∞ for SPC/E water as a function of ϵ_{CO} calculated in multiple ways. Data determined from droplet simulations is shown as blue data points. Data obtained from interfacial tensions using the full pressure tensor, as defined in Eq. (7) in the main text, with and without accounting for γ_S^{sl} as derived in Eq. (S52) are shown as yellow and red data points, respectively. Data obtained from interfacial tensions using only the virial instead of the full pressure tensor (Eq. (S57)) with and without accounting for γ_S^{sl} are shown as green and purple data points, respectively. Only using the full pressure tensor and accounting for γ_S^{sl} (yellow datapoint) gives results that agree with the droplet simulations.

References

(1) Wang, X.; Zhi, L.; Müllen, K. Transparent, Conductive Graphene Electrodes for Dye-Sensitized Solar Cells. *Nano Lett.* **2008**, *8*, 323–327.

(2) Wang, S.; Zhang, Y.; Abidi, N.; Cabrales, L. Wettability and surface free energy of graphene films. *Langmuir* **2009**, *25*, 11078–11081.

(3) Shin, Y. J.; Wang, Y.; Huang, H.; Kalon, G.; Wee, A. T. S.; Shen, Z.; Bhatia, C. S.; Yang, H. Surface-Energy Engineering of Graphene. *Langmuir* **2010**, *26*, 3798–3802.

(4) Kim, K. S.; Lee, H. J.; Lee, C.; Lee, S. K.; Jang, H.; Ahn, J. H.; Kim, J. H.; Lee, H. J. Chemical Vapor Deposition-Grown Graphene: The Thinnest Solid Lubricant. *ACS Nano* **2011**, *5*, 5107–5114.

(5) Rafiee, J.; Mi, X.; Gullapalli, H.; Thomas, A. V.; Yavari, F.; Shi, Y.; Ajayan, P. M.; Koratkar, N. A. Wetting transparency of graphene. *Nat. Mater.* **2012**, *11*, 217–222.

(6) Shih, C. J.; Wang, Q. H.; Lin, S.; Park, K. C.; Jin, Z.; Strano, M. S.; Blankschtein, D. Breakdown in the Wetting Transparency of Graphene. *Phys. Rev. Lett.* **2012**, *109*, 176101.

(7) Hsieh, C. T.; Yang, B. H.; Tzou, D. Y.; Chen, Y. F.; Chen, W. Y. Liquid repellency from graphite sheets with different oxidation levels. *Thin Solid Films* **2013**, *529*, 80–84.

(8) Kazakova, O.; Burnett, T. L.; Patten, J.; Yang, L.; Yakimova, R. Epitaxial graphene on SiC(0001): functional electrical microscopy studies and effect of atmosphere. *Nanotech.* **2013**, *24*, 215702.

(9) Li, Z.; Wang, Y.; Kozbial, A.; Shenoy, G.; Zhou, F.; McGinley, R.; Ireland, P.; Morganstein, B.; Kunkel, A.; Surwade, S. P. et al. Effect of airborne contaminants on the wettability of supported graphene and graphite. *Nat. Mater.* **2013**, *12*, 925–931.

(10) Raj, R.; Maroo, S. C.; Wang, E. N. Wettability of Graphene. *Nano Lett.* **2013**, *13*, 1509–1515.

(11) Pu, J.; Wan, S.; Lu, Z.; Zhang, G. A.; Wang, L.; Zhang, X.; Xue, Q. Controlled water adhesion and electrowetting of conducting hydrophobic graphene/carbon nanotubes composite films on engineering materials. *J. Mater. Chem. A* **2013**, *1*, 1254–1260.

(12) Ondarçuhu, T.; Thomas, V.; Nuñez, M.; Dujardin, E.; Rahman, A.; Black, C. T.; Checco, A. Wettability of partially suspended graphene. *Sci. Rep.* **2016**, *6*, 24237.

(13) Belyaeva, L. A.; van Deursen, P. M.; Barbetsea, K. I.; Schneider, G. F. Hydrophilicity of Graphene in Water through Transparency to Polar and Dispersive Interactions. *Adv. Mater.* **2018**, *30*, 1703274.

(14) Prydatko, A. V.; Belyaeva, L. A.; Jiang, L.; Lima, L. M.; Schneider, G. F. Contact angle measurement of free-standing square-millimeter single-layer graphene. *Nat. Comm.* **2018**, *9*, 4185.

(15) Morcos, I. On contact angle and dispersion energy of the cleavage graphite/water system. *J. Colloid Interf. Sci.* **1970**, *34*, 469–471.

(16) Schrader, M. E. Ultrahigh vacuum techniques in the measurement of contact angles. IV. Water on graphite (0001). *J. Phys. Chem* **1975**, *79*, 2508–2515.

(17) Schrader, M. E. Ultrahigh-Vacuum Techniques in the Measurement of Contact Angles. 5. LEED Study of the Effect of Structure on the Wettability of Graphite. *J. Phys. Chem* **1980**, *84*, 2774–2779.

(18) Ashraf, A.; Dastgheib, S. A.; Mensing, G.; Shannon, M. A. Surface characteristics of selected carbon materials exposed to supercritical water. *J. Supercrit. Fluids* **2013**, *76*, 32–40.

(19) Ashraf, A.; Wu, Y.; Wang, M. C.; Aluru, N. R.; Dastgheib, S. A.; Nam, S. Spectroscopic Investigation of the Wettability of Multilayer Graphene Using Highly Ordered Pyrolytic Graphite as a Model Material. *Langmuir* **2014**, 12827–12836.

(20) Kozbial, A.; Li, Z.; Sun, J.; Gong, X.; Zhou, F.; Wang, Y.; Xu, H.; Liu, H.; Li, L. Understanding the intrinsic water wettability of graphite. *Carbon* **2014**, *74*, 218–225.

(21) Mücksch, C.; Rösch, C.; Müller-Renno, C.; Ziegler, C.; Urbassek, H. M. Consequences of Hydrocarbon Contamination for Wettability and Protein Adsorption on Graphite Surfaces. *J. Phys. Chem. C* **2015**, *119*, 12496–12501.

(22) Fowkes, F. M.; Harkins, W. D. The State of Monolayers Adsorbed at the Interface Solid-Aqueous Solution. *J. Am. Chem. Soc.* **1940**, *62*, 3377–3386.

(23) Adamson, A. W.; Gast, A. P. *Physical Chemistry of Surfaces*, 6th ed.; John Wiley & Sons, 1997.

(24) Berendsen, H.; van der Spoel, D.; van Drunen, R. GROMACS: A message-passing parallel molecular dynamics implementation. *Comput. Phys. Commun.* **1995**, *91*, 43–56.

(25) Lindahl, E.; Hess, B.; van der Spoel, D. GROMACS 3.0: a package for molecular simulation and trajectory analysis. *J. Mol. Model.* **2001**, *7*, 306–317.

(26) Swope, W. C.; Andersen, H. C.; Berens, P. H.; Wilson, K. R. A computer simulation method for the calculation of equilibrium constants for the formation of physical clusters of molecules: Application to small water clusters. *J. Chem. Phys.* **1982**, *76*, 637–649.

(27) Bussi, G.; Donadio, D.; Parrinello, M. Canonical sampling through velocity rescaling. *J. Chem. Phys.* **2007**, *126*, 014101.

(28) Berendsen, H. J.; Grigera, J. R.; Straatsma, T. P. The missing term in effective pair potentials. *J. Phys. Chem.* **1987**, *91*, 6269–6271.

(29) Endo, M., Iijima, S., Dresselhaus, M. S., Eds. *Carbon Nanotubes*; Pergamon, 1996.

(30) Werder, T.; Walther, J. H.; Jaffe, R. L.; Halicioglu, T.; Koumoutsakos, P. On the Water–Carbon Interaction for Use in Molecular Dynamics Simulations of Graphite and Carbon Nanotubes. *J. Phys. Chem. B* **2003**, *107*, 1345–1352.

(31) Oostenbrink, C.; Villa, A.; Mark, A. E.; Van Gunsteren, W. F. A biomolecular force field based on the free enthalpy of hydration and solvation: The GROMOS force-field parameter sets 53A5 and 53A6. *J. Comput. Chem.* **2004**, *25*, 1656–1676.

(32) Darden, T.; York, D.; Pedersen, L. Particle mesh Ewald: An $N \cdot \log(N)$ method for Ewald sums in large systems. *J. Chem. Phys.* **1993**, *98*, 10089–10092.

(33) Kanduč, M.; Eixeres, L.; Liese, S.; Netz, R. R. Generalized line tension of water nanodroplets. *Phys. Rev. E* **2018**, *98*, 032804.

(34) Carlson, S.; Becker, M.; Brünig, F. N.; Ataka, K.; Cruz, R.; Yu, L.; Tang, P.; Kanduč, M.; Haag, R.; Heberle, J. et al. Hydrophobicity of Self-Assembled Monolayers of Alkanes: Fluorination, Density, Roughness, and Lennard-Jones Cutoffs. *Langmuir* **2021**, *37*, 13846–13858.

(35) Wennberg, C. L.; Murtola, T.; Hess, B.; Lindahl, E. Lennard-Jones Lattice Summation in Bilayer Simulations Has Critical Effects on Surface Tension and Lipid Properties. *9*, 3527–3537, Publisher: American Chemical Society.

(36) Jorgensen, W. L.; Chandrasekhar, J.; Madura, J. D.; Impey, R. W.; Klein, M. L. Comparison of simple potential functions for simulating liquid water. *J. Chem. Phys.* **1983**, *79*, 926–935.

(37) Izadi, S.; Onufriev, A. V. Accuracy limit of rigid 3-point water models. *J. Chem. Phys.* **2016**, *145*, 074501.

(38) Horn, H. W.; Swope, W. C.; Pitera, J. W.; Madura, J. D.; Dick, T. J.; Hura, G. L.; Head-Gordon, T. Development of an improved four-site water model for biomolecular simulations: TIP4P-Ew. *J. Chem. Phys.* **2004**, *120*, 9665–9678.

(39) Abascal, J. L. F.; Vega, C. A general purpose model for the condensed phases of water: TIP4P/2005. *J. Chem. Phys.* **2005**, *123*, 234505.

(40) Izadi, S.; Anandakrishnan, R.; Onufriev, A. V. Building Water Models: A Different Approach. *J. Phys. Chem. Lett.* **2014**, *5*, 3863–3871.

(41) Rick, S. W. A reoptimization of the five-site water potential (TIP5P) for use with Ewald sums. *J. Chem. Phys.* **2004**, *120*, 6085–6093.

(42) Kanduč, M.; Netz, R. R. From hydration repulsion to dry adhesion between asymmetric hydrophilic and hydrophobic surfaces. *Proc. Nat. Acad. Sci.* **2015**, *112*, 12338–12343.

(43) Sega, M.; Fábián, B.; Jedlovszky, P. Nonzero Ideal Gas Contribution to the Surface Tension of Water. *J. Phys. Chem. Lett.* **2017**, *8*, 2608–2612.

(44) Sedlmeier, F.; Janecek, J.; Sendner, C.; Bocquet, L.; Netz, R. R.; Horinek, D. Water at polar and nonpolar solid walls (Review). *Biointerphases* **2008**, *3*, FC23–FC39.

(45) Sendner, C.; Horinek, D.; Bocquet, L.; Netz, R. R. Interfacial Water at Hydrophobic and Hydrophilic Surfaces: Slip, Viscosity, and Diffusion. *Langmuir* **2009**, *25*, 10768–10781.
